# The rise of macropredatory pliosaurids near the Early-Middle Jurassic transition

**DOI:** 10.1038/s41598-023-43015-y

**Published:** 2023-10-16

**Authors:** Sven Sachs, Daniel Madzia, Ben Thuy, Benjamin P. Kear

**Affiliations:** 1 Abteilung Geowissenschaften, Naturkunde-Museum Bielefeld, Adenauerplatz 2, 33602 Bielefeld, Germany; 2 Institute of Paleobiology, Polish Academy of Sciences, Twarda 51/55, 00818 Warsaw, Poland; 3 Department of Palaeontology, Natural History Museum Luxembourg, 25, rue Münster, 2160 Luxembourg City, Luxembourg; 4 The Museum of Evolution, Uppsala University, Norbyvägen 16, 752 36 Uppsala, Sweden

**Keywords:** Palaeontology, Phylogenetics, Taxonomy, Evolutionary ecology

## Abstract

The emergence of gigantic pliosaurid plesiosaurs reshaped the trophic structure of Mesozoic marine ecosystems, and established an  ~ 80 million-year (Ma) dynasty of macropredatory marine reptiles. However, the timescale of their ‘defining’ trait evolution is incompletely understood because the fossil record of gigantic pliosaurids is scarce prior to the late-Middle Jurassic (Callovian),  ~ 165.3 Ma. Here, we pinpoint the appearance of large body size and robust dentitions to early-Middle Jurassic (Bajocian) pliosaurids from northeastern France and Switzerland. These specimens include a new genus that sheds light on the nascent diversification of macropredatory pliosaurids occurring shortly after the Early-Middle Jurassic transition, around  ~ 171 Ma. Furthermore, our multivariate assessment of dental character states shows that the first gigantic pliosaurids occupied different morphospace from coeval large-bodied rhomaleosaurid plesiosaurs, which were dominant in the Early Jurassic but declined during the mid-Jurassic, possibly facilitating the radiation and subsequent ecomorph acme of pliosaurids. Finally, we posit that while the emergence of macropredatory pliosaurids was apparently coordinated with regional faunal turnover in the epeiric basins of Europe, it paralleled a globally protracted extinction of other higher trophic-level marine reptiles that was not completed until after the earliest-Late Jurassic,  ~ 161.5 Ma.

## Introduction

The pliosaurid plesiosaur (Pliosauridae, Plesiosauria) clade Thalassophonea, or ‘sea murderers’, encompassed a taxonomically diverse marine reptile lineage whose fossils have been identified from Middle Jurassic to lower–Upper Cretaceous strata virtually worldwide^1–6^. The group was characterised by proportionally very large skulls, robust dentitions, short necks, and mega-body size with some forms exceeding lengths of 10 m^7–9^. However, the evolutionary timescale over which thalassophonean pliosaurids acquired these apex-predator traits is contentious because substantial gaps exist in the plesiosaur fossil record^10^. Indeed, although the origin of Thalassophonea has been estimated to occur near the Early-to-Middle Jurassic transition^11^, there are as yet no definitively attributable skeletal remains recognised from strata older than Callovian (upper-Middle Jurassic). Moreover, with the exception of the lower Callovian *Marmornectes candrewi*^12^ and middle Oxfordian (Upper Jurassic) *Anguanax zignoi*^13^, all other more basally divergent non-thalassophonean pliosaurids are Early Jurassic in age, and typified by comparatively small skulls, gracile dentitions, long necks, and diminutive body sizes (up to ~ 4 m)^14–19^.

**Figure 1:**
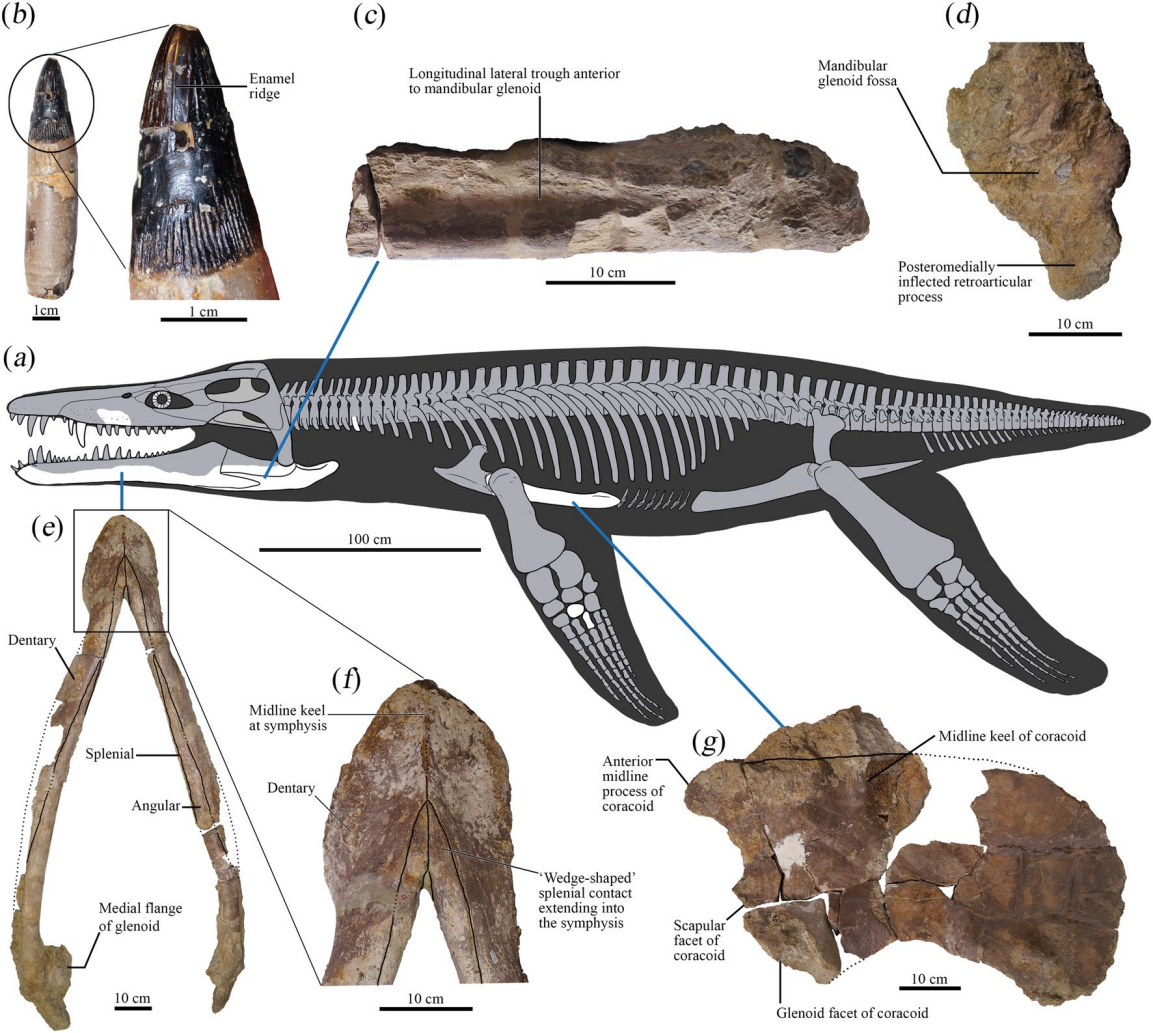
Skeletal remains of the holotype (MNHNL BU159) of *Lorrainosaurus keileni*. (**a**) Reconstruction in lateral view showing recovered elements. (**b**) Tooth crown with root. (**c**) Posterior section of mandible in lateral view. (**d**) Glenoid section of mandible in articular view. (**e**) Complete mandible in ventral view. (**f**) Enlargement of the mandibular symphysis. (**g**) Coracoid in dorsal view.

Here, we assess one of the geologically oldest unequivocal thalassophonean pliosaurids from the upper Bajocian (mid-Middle Jurassic) Marnes de Gravelotte of Lorraine in northeastern France. This taxon is represented by a partial skeleton (Fig. 1a) excavated in 1983 by palaeontology enthusiasts from the Association minéralogique et paléontologique d’Hayange et des environs (AMPHE). The fossils were later donated to the Musée national d’histoire naturelle de Luxembourg (MNHNL) and identified as a species of the Callovian pliosaurid *Simolestes*^20^. Although since only briefly mentioned in the literature, this taxon—‘*Simolestes*’ *keileni* (Godefroit, 1994)^20^—is significant because the type specimen (MNHNL BU159) preserves an almost complete mandible that is 1.33 m in maximum length and incorporates a robust dentition indicative of macropredatory thalassophoneans. MNHNL BU159 is also both geographically and stratigraphically proximal to a second very large (~ 1.5 m in maximum length) but incomplete “pliosaurid-like” mandible (Paläontologisches Institut und Museum der Universität Zürich [PIMUZ] A/III0521) that was recovered from the lower Bajocian Passwang Formation near Arisdorf in the Basel-Land canton of Switzerland^21^.

‘*Simolestes*’ *keileni* and PIMUZ A/III0521 have thus been advocated as “evidence for the continuous presence of gigantic apex predatory plesiosaurs in Europe” following a marine reptile turnover in western Europe (Fischer et al.^22^, p. 28) that spanned the Aalenian (earliest-Middle Jurassic), and was marked, among others, by the replacement of Early Jurassic large-bodied rhomaleosaurid (Rhomaleosauridae) plesiosaurs by archetypal later Jurassic pliosaurids that then dispersed globally as the highest trophic-level predators^22^. Notably, though, no unambiguous pliosaurid fossils were actually identified from these studied successions, and the systematic affinities of ‘*S*’. *keileni* and PIMUZ A/III0521 have hitherto remained uncertain.

We provide detailed osteological description of ‘*S*’. *keileni* and perform phylogenetic and multivariate analyses (Electronic Supplementary Material 1–5), targeting ‘*S*’. *keileni* and PIMUZ A/III0521, to: (1) establish the phylogenetic placement of the specimens within Plesiosauria; (2) compare the dental morphospace occupation of ‘*S*’. *keileni* with that of pliosaurid and rhomaleosaurid plesiosaurs; and (3) explore the timescale and global context of plesiosaur apex-predator turnover associated with the Early-to-Middle Jurassic transition.

### Institutional abbreviations

GFMSU, Geological Faculty, Lomonosov Moscow State University, Moscow, Russian Federation; GIK, Institut für Geologie und Mineralogie, Universität Köln, Cologne, Germany; MNHNL, Musée national d’histoire naturelle de Luxembourg, Luxembourg; MWGUW, Stanisław Józef Thugutt Geological Museum, Faculty of Geology, University of Warsaw, Warsaw, Poland; NNGASU, Museum of Nizhny Novgorod State University of Architecture, Nizhny Novgorod, Russian Federation; PIMUZ, Paläontologisches Institut und Museum der Universität Zürich, Zürich, Switzerland; SOIKM, Samara Regional History and Local Lore Museum named after P. V. Alabin, Samara, Russian Federation; TsNIGR, Central Scientific Research Geological Survey Museum named after Academician F. N. Chernyshev, St Petersburg, Russian Federation.

## Geological and stratigraphic setting

**Figure 2:**
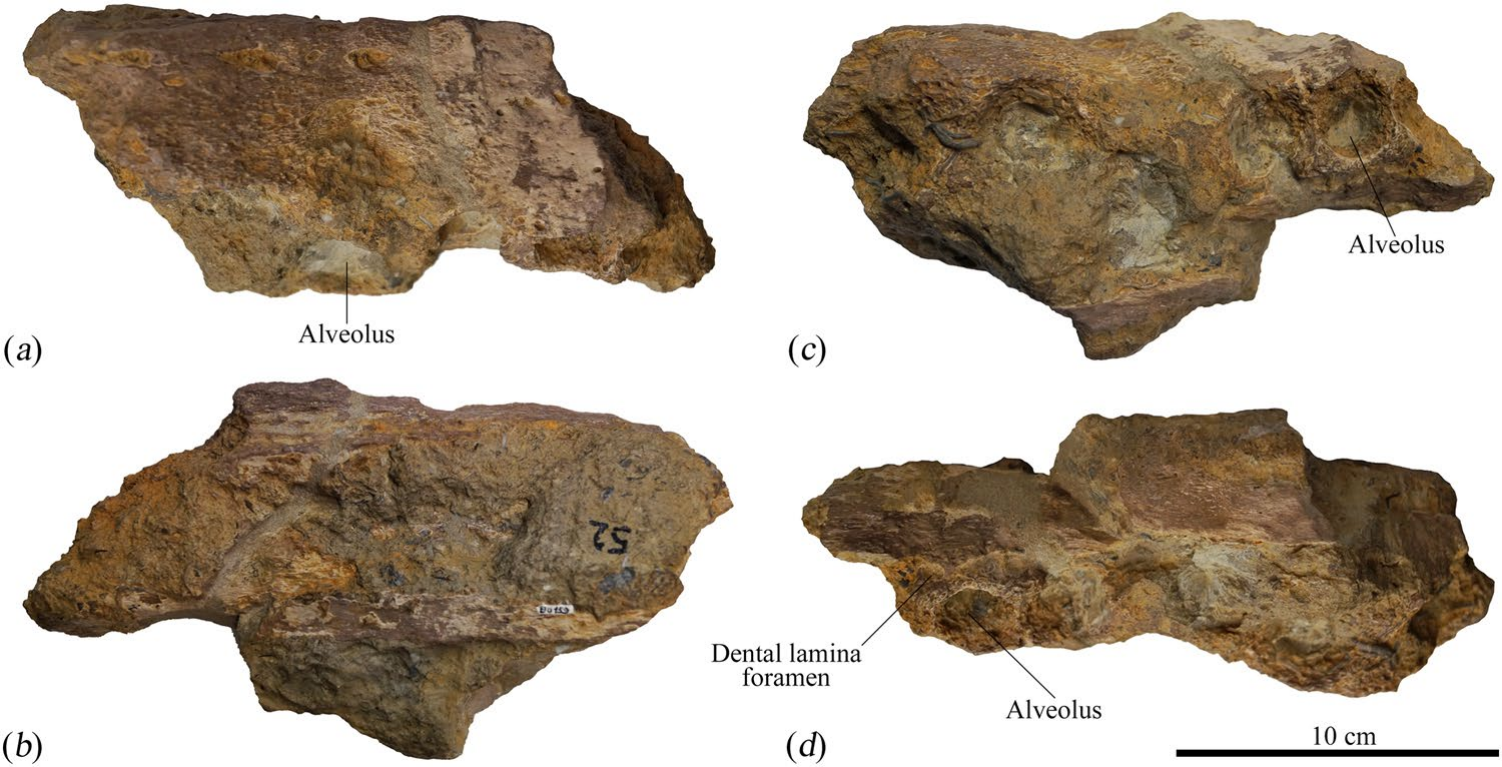
Maxilla fragment from the holotype (MNHNL BU159) of *Lorrainosaurus keileni* in (**a**) lateral, (**b**) dorsal, (**c**) ventral, and (**d**) medial views.

**Figure 3:**
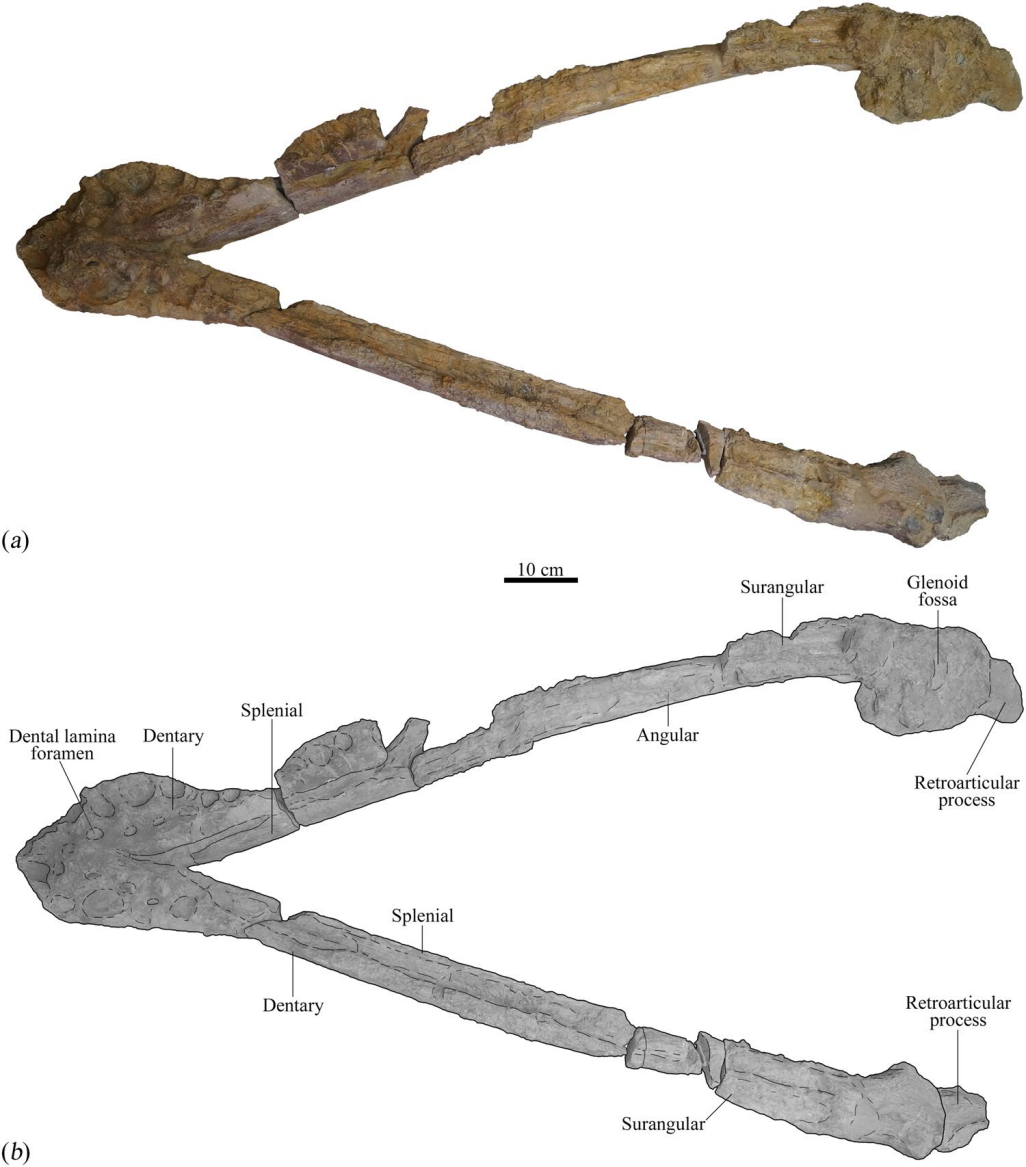
Mandible from the holotype (MNHNL BU159) of *Lorrainosaurus keileni* in dorsal view: (**a**) photograph; (**b**) graphic illustrating individual bones and important structures.

**Figure 4:**
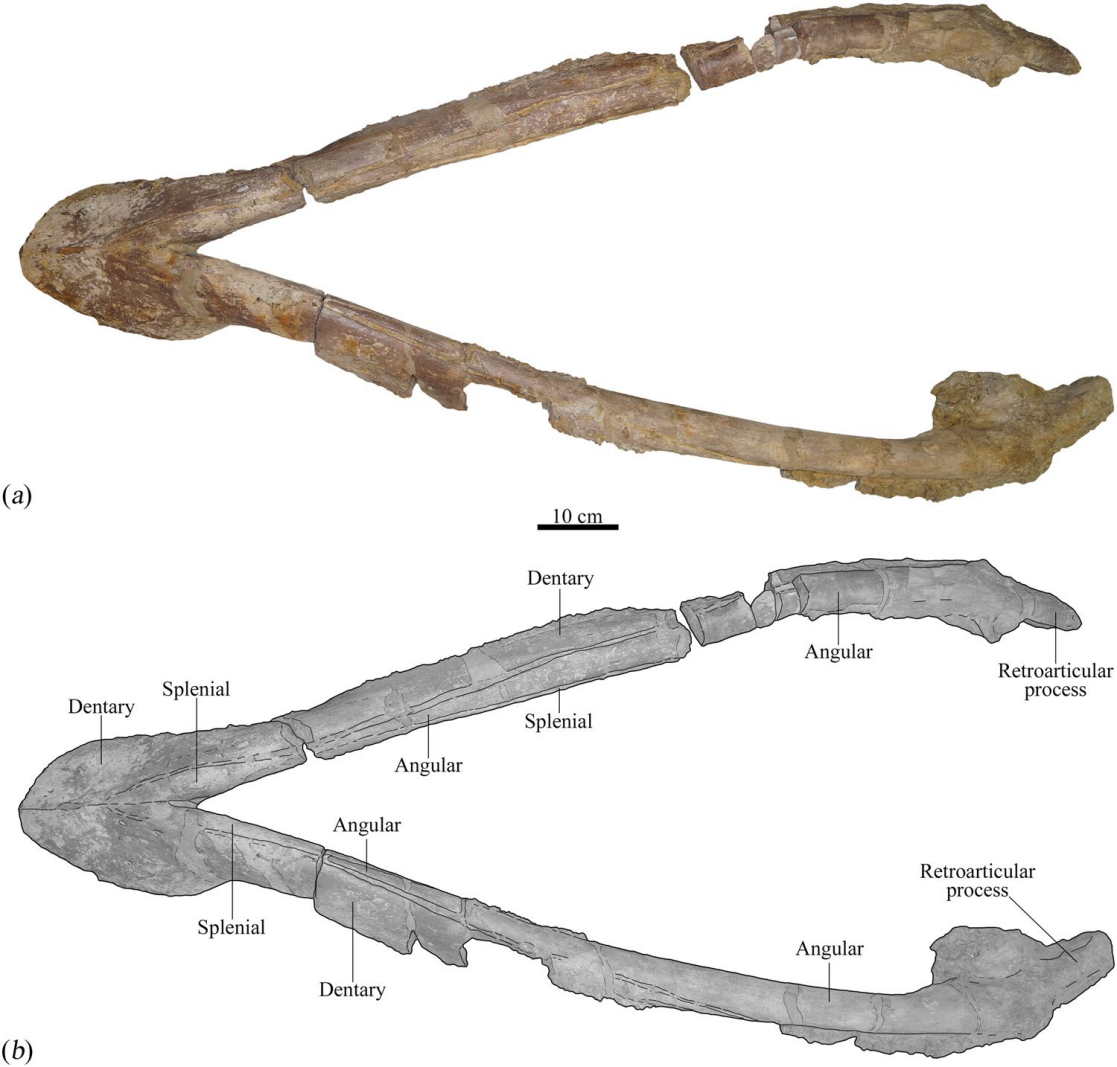
Mandible from the holotype (MNHNL BU159) of *Lorrainosaurus keileni* in ventral view: (**a**) photograph; (**b**) graphic illustrating individual bones and important structures.

**Figure 5:**
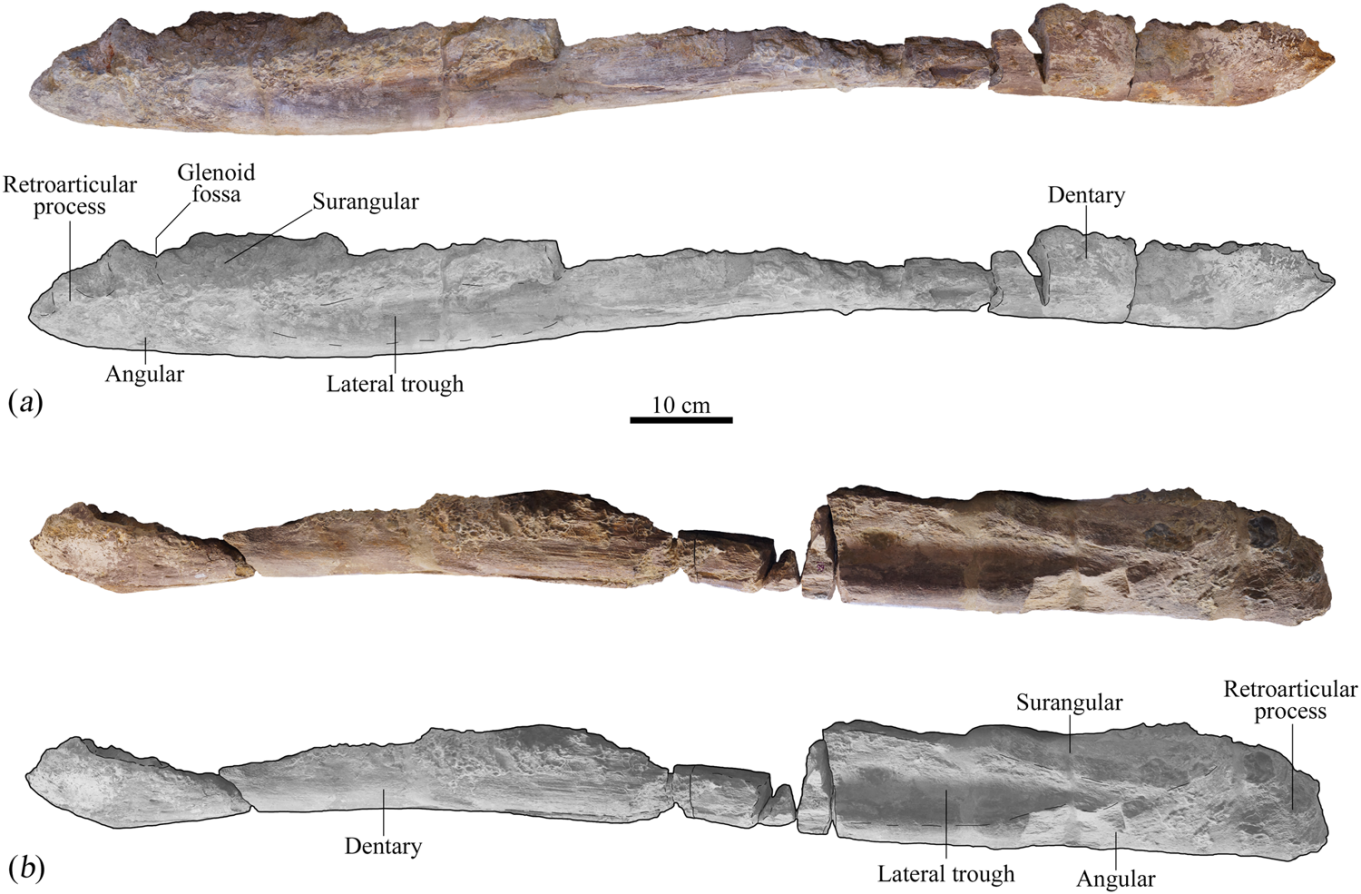
Mandible from the holotype (MNHNL BU159) of *Lorrainosaurus keileni* in right (top) and left (bottom) lateral views: (**a**) photograph; (**b**) graphic illustrating individual bones and important structures.

**Figure 6:**
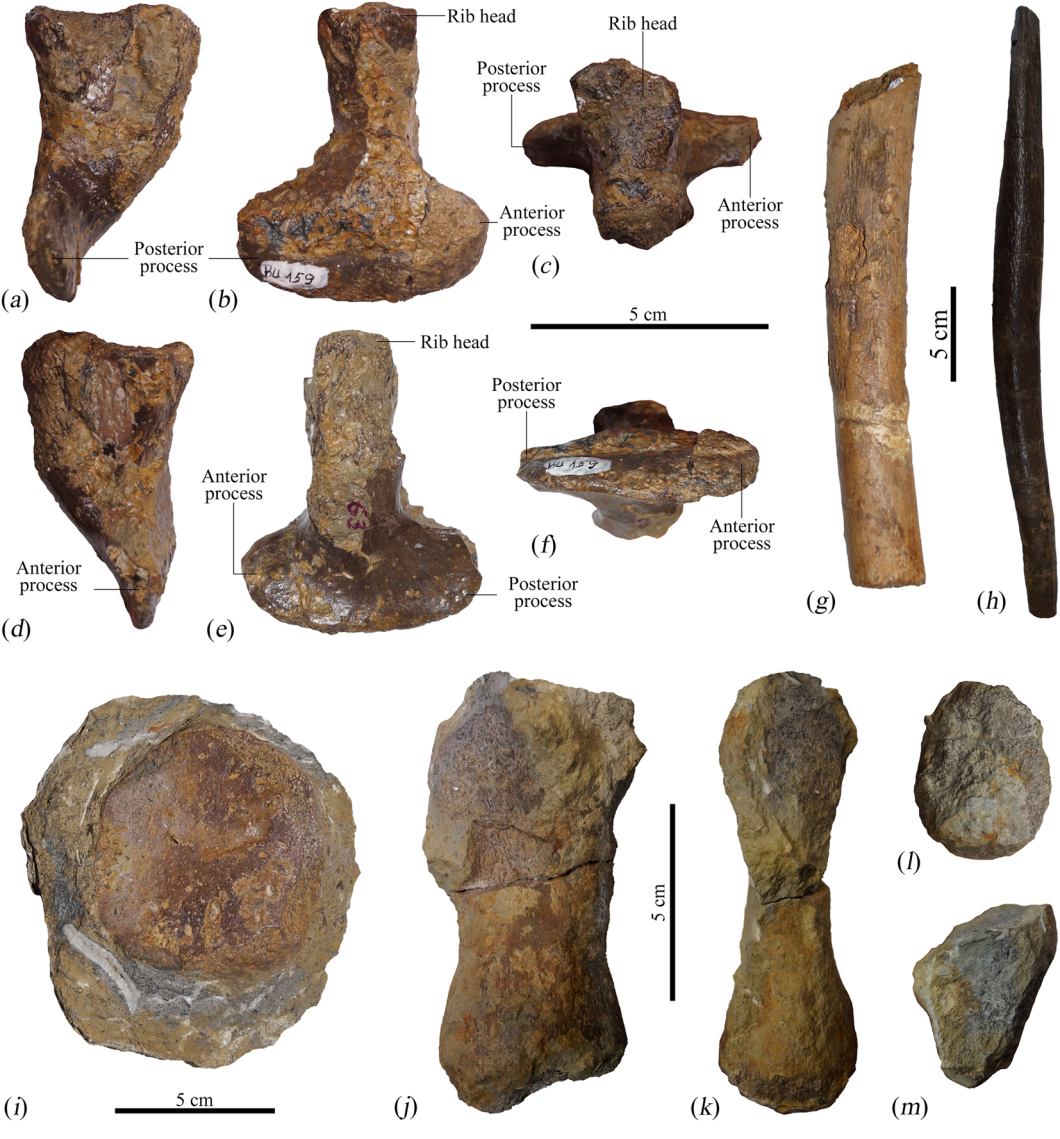
Postcranial elements from the holotype (MNHNL BU159) of *Lorrainosaurus keileni*. Left cervical rib in (**a**) posterior, (**b**) lateral, (**c**) dorsal, (**d**) anterior, (**e**) medial, and (**f**) ventral views. (**g**) Dorsal rib section. (**h**) Gastral rib. (**i**) Probable mesopodial element in dorsoventral view. Phalanx in (**j**) dorsoventral, (**k**) anteroposterior, (**l**) proximal, and (**m**) ventral views.

**Figure 7:**
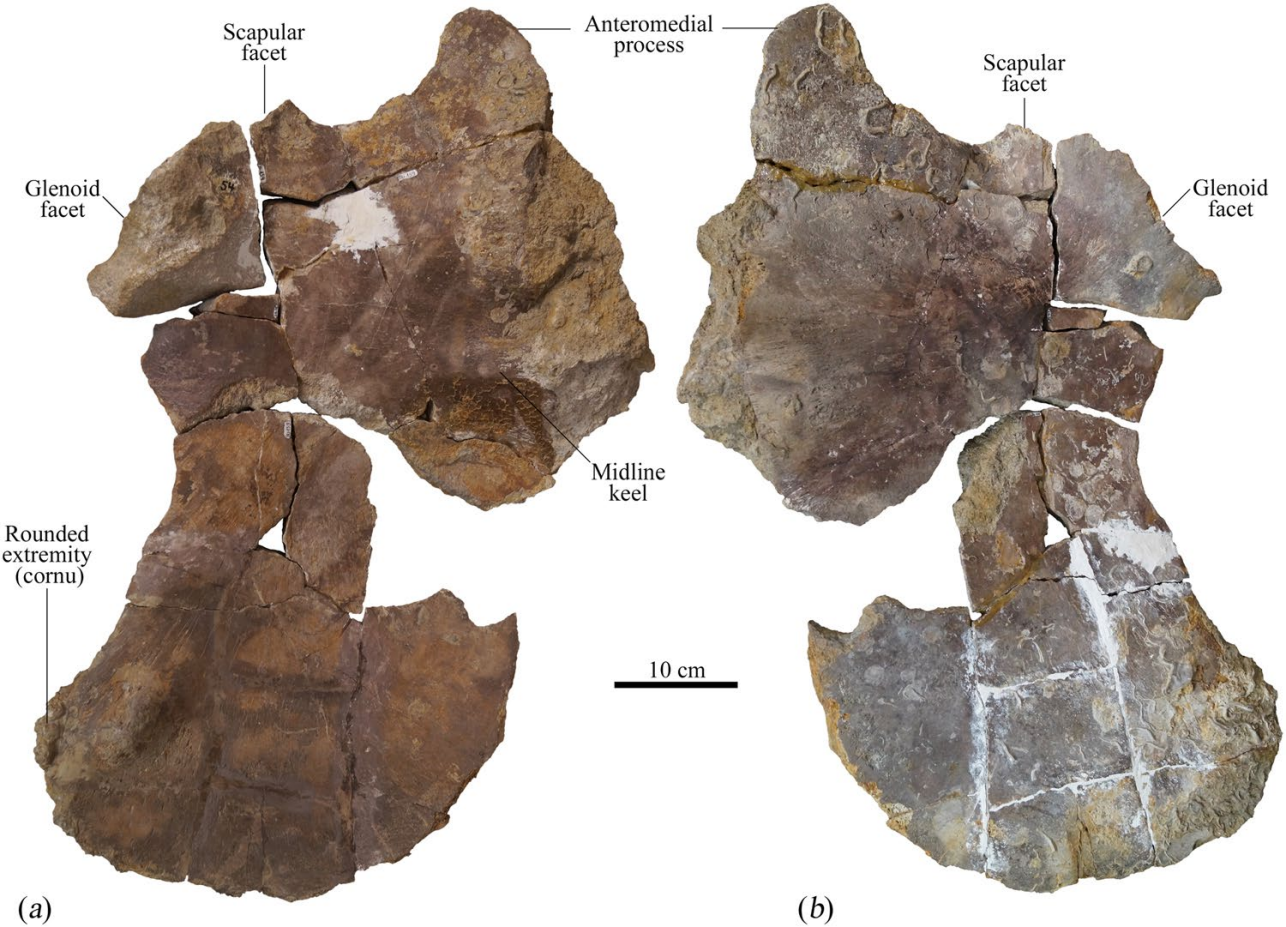
Left coracoid from the holotype (MNHNL BU159) of *Lorrainosaurus keileni* in (**a**) dorsal, and (**b**) ventral views.

MNHNL BU159 was recovered from a temporary cutting excavated during an upgrade of the roadway between Montois-la-Montagne and Sainte-Marie-aux-Chênes near Metz in Lorraine, northeastern France^20^ (Electronic Supplementary Material 6, Fig. S1). At the time of discovery, this exposure revealed a succession of grey-blue marls and yellow–brown argillaceous limestones that were rich in shell debris. The skeletal remnants of MNHNL BU159 (Figs. 2, 3, 4, 5, 6 and 7, Electronic Supplementary Material 6, Figs S2–S4) occurred within a sandy calcareous marl horizon equivalent to the Marnes de Gravelotte^23,24^, a regional lithostratigraphical unit that extends along the northeastern margin of the Paris Basin. The Marnes de Gravelotte is laterally and stratigraphically bounded by oolithic limestones and corresponds to a localised siliciclastic episode that affected marine sediment accumulation along the northeastern periphery of the Middle Jurassic Burgundy carbonate platform^24^.

The Marnes de Gravelotte has been specifically correlated with the upper Bajocian *Parkinsonia parkinsoni* Zone, and yields abundant marine invertebrate assemblages comprising epifaunal and infaunal bivalves, belemnites, ammonites, terebratulid and rhynchonellid brachiopods, echinoderms, and serpulid annelids^24^. The depositional setting is interpreted as subtidal and above storm-wave base with episodic high-energy conditions^24^. The seafloor seems to have been well-oxygenated and intensely bioturbated, as exemplified by the bones of MNHNL BU159, which exhibit extensive bio-encrustation by serpulids and bivalves. This is most prolific on the dentigerous surfaces of the mandible and ventral surface of the coracoid, but also affects the gastral ribs. We interpret this as evidence for the skeletal remnants of MNHNL BU159 having decayed, dispersed and lain exposed on the seafloor for some time where they served as hard substrate islands for benthic invertebrates before eventually becoming buried in muddy substrate.

## Material and methods

### Plesiosaur dental terminology

Our terminology for the anatomical orientation of plesiosaur teeth refers to ‘apical’ as the direction toward the tooth crown apex, and ‘basal’ describing the direction towards the *cervix dentis*^25^. Likewise, ‘distal’ and ‘mesial’ indicate the directions either away from or towards the snout tip, respectively. Lastly, ‘labial’ implies the direction towards the lips, and ‘lingual’ implies the direction towards the tongue. Plesiosaur dental enamel surface morphology complies with other recent studies^3,5,26–32^ in defining ‘apicobasal ridges’ as extending longitudinally from the crown apex to base, and also usually being semicircular or triangular in cross-section. In turn, ‘ridglets’ refers to subtle apicobasally-oriented enamel structures often developed between adjacent apicobasal ridges or on the non-ridged enamel surface; ‘ridglets’ can also be smooth or developed into a vermiculate surface ornamentation (Madzia^32^, Fig. 7).

### Phylogenetic analyses

The phylogenetic relationships of pliosaurid plesiosaurs were investigated using the dataset of Sachs et al.^33^. We obtained first-hand scores for MNHNL BU159 and PIMUZ A/III0521, and also added information for the Early Jurassic (late Pliensbachian) pliosaurids *Arminisaurus schuberti*^19^ and *Cryonectes neustriacus*^18^, and for the Middle Jurassic (middle Callovian) thalassophonean *Eardasaurus powelli*^34^, based on original observations, photographs, and the literature. Our TNT 1.6^35,36^ search methods utilised an ordered ‘*ccode*’ set, with (1) an unweighted parsimony analysis (UPWa); (2) a weighted parsimony analysis (IWa) with *K* = 6; (3) IWa with the *K*-value selected so that the weight ratio between no homoplasy and maximum possible steps was 1 to 10 (default value); and (4) enforced monophyly of MNHNL BU159 and *Simolestes vorax* to assess the sister-taxon relationship between the two operational taxonomic units (OTUs) as indicated by original assignment of MNHNL BU159 to *Simolestes*^20^. Maxtrees were manually fixed at ‘hold 200000;’. Our initial ‘New Technology’ search involved 1000 addition sequences and default settings activated for sectorial searches, ratchet, drift, and tree fusing. A subsequent ‘Traditional Search’ with tree bisection-reconnection (TBR) branch-swapping was performed on trees saved to RAM. Bremer support was calculated for UPWa with TBR and sub-optimal trees retained with up to three additional steps. Node support was determined using Symmetric Resampling for IWa with a ‘Traditional’ search, 1000 replicates, the default change probability P = 33, and output expressed as GC frequency differences.

Tree topologies and numerical results from our phylogenetic analyses, as well as our character state matrix are presented in Electronic Supplementary Material 1 and 2.

### Multivariate analyses

We added MNHNL BU159 to the pliosaurid dental character state dataset of Zverkov et al.^3^, but with a corrected carinal score (character 3) for the ‘Crimean pliosaurid’ GFMSU h-216 (0 → 2)^3, 5, 37^, and new information on selected rhomaleosaurid plesiosaurs (Electronic Supplementary Material 3). Our modified matrix (Electronic Supplementary Material 3) was subjected to a 50% completeness threshold to mitigate the effects of missing state entries. The data were also scaled to equal variance and a zero mean applied through subtraction of the mean value for each character divided by the standard deviation. Using the cluster 2.1.0 package in *R*^38^ we applied the Gower metric^39^ to create a distance matrix. Our cluster dendrograms were produced using the stats package and the Ward.D2 method. Our principal coordinates analysis (PCoA) was undertaken with the ape 5.3 package^40^ and a Gower metric with Cailliez correction for negative eigenvalues. All *R* code is supplied in the Electronic Supplementary Material 4.

### Nomenclatural acts

This published work and the nomenclatural acts it contains have been registered in ZooBank, the proposed online registration system for the International Code of Zoological Nomenclature (ICZN). The ZooBank LSIDs (Life Science Identifiers) can be resolved and the associated information viewed through any standard web browser by appending the LSIDs to the prefix http://zoobank.org/. The LSIDs are urn:lsid:zoobank.org:pub:5CF1780F-3728- 423C-A1E5-111DE436F2D0 for this publication and urn:lsid:zoobank.org:act:1ED4F59D-AEAB-4142-872C- 2B974FFD8D05 for the new genus *Lorrainosaurus*.

## Systematic palaeontology

Plesiosauria de Blainville, 1835^41^

Pliosauridae Seeley, 1874^42^

Thalassophonea Benson & Druckenmiller, 2014^1^

*Lorrainosaurus* gen. nov.

### Etymology

Derived from ‘Lorraine’, for the type locality; and ‘*σαῦρος*’ (*sauros*), Greek for ‘reptile’.

### Type species

*Lorrainosaurus keileni* (Godefroit, 1994)^20^.

### Holotype

MNHNL BU159, an incomplete skeleton (Fig. 1a) comprising four associated teeth (Fig. 1b), a maxilla fragment and articulated mandible (Fig. 1a–f), together with a left cervical rib and dorsal and gastral rib sections, a mesopodial element and phalanx, the left coracoid (Fig. 1g), and several other unidentifiable bone remnants (Figs. 2, 3, 4, 5, 6 and 7, Electronic Supplementary Material 6, Figs S2–S5).

### Type locality and stage

A temporary road cutting between Montois-la-Montagne and Sainte-Marie-aux-Chênes ~ 18 km northeast of Metz in Lorraine, northeastern France^20^. These deposits form part of the Marnes de Gravelotte regional lithostratigraphical unit correlated with the upper Bajocian (mid-Middle Jurassic) *Parkinsonia parkinsoni* Zone.

### Diagnosis

Large-bodied thalassophonean pliosaurid autapomorphically distinguished by a transversely broad, ‘wedge-shaped’ splenial contact that extends anteriorly to the level of the fourth mandibular alveolus. *Lorrainosaurus keileni* also displays a unique character state combination: (1) laterally expanded and posteriorly constricted ‘spatulate’ symphyseal section of the mandible bearing five to six alveoli; (2) lateral trough on the mandible anterior to the glenoid fossa; (3) a retroarticular process that is shorter than the glenoid fossa; (4) retroarticular process with posteroventrally oriented dorsoventral long axis and slightly posteromedially inflected mediolateral long axis; (5) wide posteromedial seperation of the coracoids; (6) posterolateral edge of the coracoid (cornu) projecting beyond the level of the glenoid fossa (Fig. 1).

### Description and comparisons of MNHNL BU159

#### Cranium and mandible

MNHNL BU159 comprises an incomplete skeleton that was apparently disarticulated and dispersed prior to burial. The cranium was not described by Godefroit^20^, but is represented by a section of the maxilla that preserves at least four discernible alveoli, but potentially has up to six tooth positions accommodated in succession (Fig. 2).

The alveoli (herein designated MA1–MA4, Electronic Supplementary Material 6, Fig. S3a) are 21–24 mm in maximum diameter and upright rather than procumbent, suggesting an original placement towards the posterior maxillary tooth row (based on comparisons with complete dentitions attributed to *Pliosaurus*^43^. However, MA2 is offset out of alignment, and is thus reminiscent of the transversely constricted mid-maxillary tooth row in *Liopleurodon ferox* (Andrews^44^, p. 6, Text-Fig. 1). Indeed, the finished exterior bone surface is perforated by sparse foramina and has an undulating profile that expands laterally around the alveoli, but is constricted by a vertical trough that likely bordered a diastema between MA2–MA3. The medial and dorsal surfaces of the maxilla fragment are damaged, with the dorsal surface having suffered severe corrosion, possibly through abrasion during transport and subsequent bioerosion on the seafloor.

In contrast to the cranium, the mandible of MNHNL BU159 is largely intact with a well-preserved symphyseal section (Electronic Supplementary Material 6, Fig. S2) and exterior bone surfaces extending posterior to the retroarticular processes (Figs. 3, 4 and 5, Electronic Supplementary Material 6, Fig. S4). Godefroit (^20^, p. 86) identified up to six alveoli in the symphyseal part of the mandible with the first and sixth being smallest and possessing a diagnostic “forme elliptique”. We alternatively interpret the alveolar shape as being more irregularly oval to circular in outline, with five and a half symphyseal alveoli (designated mA1–mA6) discernible on the right mandibular ramus, and at least five on the left (Electronic Supplementary Material 6, Figs S2a, S3b).

This arrangement compares with *S. vorax*, ‘*Polyptychodon*’ *hudsoni*, and *Acostasaurus pavachoquensis*, which also have five–six symphyseal tooth positions^44–46^. Conversely, only three–four tooth positions parallel the symphysis in *Sachicasaurus vitae*^4^, and the basally branching pliosaurids *Thalassiodracon hawkinsii*^15,43^ and *Stratesaurus taylori*^47^.

Symphyses with six–seven tooth positions are otherwise typical of pliosaurids, such as *L. ferox*, *Brachauchenius lucasi*, *Pliosaurus macromerus*, ‘*Pliosaurus*’ *rossicus*, *Megacephalosaurus eulerti*, *Cryonectes neustriacus* and *Kronosaurus queenslandicus*^18,44,48–51^. *Pliosaurus brachydeirus*, *Pliosaurus brachyspondylus*, and *Pliosaurus carpenteri*^49,52^ alternatively have eight–nine symphyseal tooth positions, with ‘*Pliosaurus*’ *andrewsi*^53^, *Marmornectes candrewsi*^12^, *Stenorhynchosaurus munozi*^4^, and probably *Makhaira rossica*^2^ having 10–12, and *Peloneustes philarchus* possessing 14–16^44^.

The symphyseal section of the mandible in MNHNL BU159 is transversely expanded (180 mm in maximum transverse width) and ‘spatulate’^54^, accommodating for a progressive increase in alveolar size up to the dimensionally largest mA4–mA5 tooth positions (37–39 mm in maximum diameter, respectively). The alveolar diameter subsequently decreases from mA6 (31 mm in maximum diameter) to mA7–mA8, which are the smallest alveoli (22–20 mm in maximum diameter, respectively) and situated within a tapered “dentary constriction” (Gómez-Pérez & Noè^46^, p. 25),  ~ 175 mm from the rostral-most tip of the dentary. Laterally expanded and posteriorly constricted mandibular symphyses are historically characteristic of the genus *Simolestes*, including *S. vorax*^44^ and *Simolestes indicus*^54^, but also typify some Early Cretaceous pliosaurids, such as *A. pavachoquensis*^46^, as well as the indeterminate ‘short-necked’ plesiosaur from the Berriasian (lowermost Cretaceous) of northwestern Germany (e.g. GIK 2120^55^), and the Early–Middle Jurassic rhomaleosaurids *Rhomaleosaurus thorntoni*^56^, *Maresaurus coccai*^57^, *Meyerasaurus victor*^58^, *Atychodracon megacephalus*^59^, and *Thaumatodracon wiedenrothi*^60^. Holland^51^ further noted that marginal “embayments” accommodated the overhanging premaxillary teeth in *S. vorax* and some other pliosaurids (e.g. *K. queenslandicus*^51^, p. 7, Fig. 5); however, these are not evident in MNHNL BU159 where the symphyseal margin is smoothly rounded.

The symphyseal alveoli of MNHNL BU159 are procumbent and dorsolaterally inclined in the right ramus, but seem to be distorted and damaged on the left. Benson et al.^49^ otherwise considered dorsolateral orientation of the symphyseal alveoli to be an autapomorphy of* Pliosaurus kevani*.

Medially, the symphyseal alveoli of MNHNL BU159 are paralleled by a series of dental lamina foramina^61^ that house remnants of at least three replacement teeth (Electronic Supplementary Material 6, Fig. S2a). There is also a conspicuous “fossa” or “vacuity” (sensu^59,62^) along the inset midline suture that may have connected with openings to the Meckelian canal as described in *S. vorax*^63^, *A. pavachoquensis*^46^, *P. philarchus*^64^, *Hauffiosaurus tomistomimus*^16^, and *P. kevani*^49^, as well as the rhomaleosaurids *R. thorntoni*^56^, *A. megacephalus*^59^, *T. wiedenrothi*^60^, and the indeterminate ‘short-necked’ plesiosaur from the Berriasian of Germany^55^.

Externally, the mandibular symphysis of MNHNL BU159 encloses an autapomorphically broad and ‘wedge-shaped’ splenial contact that extends anteriorly up to the level of mA4. The splenial also forms a projecting platform along the symphyseal midline that lacks any obvious contribution from the coronoid as occurs in *B. lucasi*, *P. philarchus*, *P. kevani*, and *K. queenslandicus*^48,49,51,64^; both the coronoid and angular contact the mandibular symphysis in *Pliosaurus almanzaensis*^65^. Strikingly similar “wide ventral ridge[s]” have been illustrated on the mandibular symphyses of the rhomaleosaurids *A. megacephalus* (Smith^59^, p. 6, Fig. 3) and *Macroplata tenuiceps* (Ketchum & Smith^66^, p. 1072, Fig. 1), but these do not integrate the splenial as a prominent ‘wedge-shaped’ element.

The external surfaces of the dentary are perforated by numerous small nutrient foramina (Electronic Supplementary Material 6, Fig. S2b,c). Foffa et al.^67^ showed that such foramina connect to intra-osseous channels that potentially housed a dermal sensory system. Similar interpretations have been proposed for the mandibular channels in ichthyosaurs^68^, and might evince crocodilian-like pressure receptors^69^, or electroreceptors as in some aquatic mammals (e.g. dolphins)^70^.

Most of the post-symphyseal alveolar row has been lost to weathering, although a sequence of four–five anterior alveoli (mA9–mA13) are still preserved on the right mandibular ramus (Fig. 3). The most complete of these (mA11–mA12) are 31–27 mm in maximum diameter, respectively, suggesting that the dentition was anisodont^43,71^, with the largest functional teeth situated in the rostral-most section of the jaw around tooth positions mA4–mA5. Andrews^44^ reported an identical tooth-size distribution in *S. vorax*, and anisodont dentitions also occur in species of *Pliosaurus*^43^, *L. ferox*^63^, *A. pavachoquensis*^46^, *Monquirasaurus boyacensis*^72^ and *K. queenslandicus*^51^.

Godefroit (^20^, p. 80) suggested that a “profonde encoche” (= “deep notch”) along the exposed post-symphyseal edge of the splenial articulated with the coronoid, although this could not be confirmed. On the other hand, the angular clearly extends along the entire length of the mandible and underlaps the retroarticular process; anteriorly the angular intercalates between the splenial and dentary behind the symphyseal confluence (Fig. 3).

None of the proximal mandibular elements have traceable sutures, but remnants of the surangular, angular, prearticular (extending posteriorly below the glenoid as in *H. tomistomimus*^16^, and articular all appear to be in life-position (Fig. 5 and Electronic Supplementary Material 6, Fig. S4a). The mandibular fossae are badly damaged and the coronoid processes, together with most of the surangular and dentary have eroded away to expose the floors of the Meckelian canals (Fig. 3a).

The medial surfaces of the mandible are not visibly fenestrated in the post-symphyseal region (e.g. as in *A. pavachoquensis*^46^), nor near the posterior mandibular foramen^46^. Like other pliosaurids (Ketchum & Benson^73^, appendix 3, character 102), the glenoid fossa is dorsomedially inclined and projects medially as a prominent flange (130 mm in maximum width); this imparts a distinctively “bowed” (Druckenmiller & Russell^74^, p. 43, character 75) mandibular profile in occlusal view (Fig. 3 and Electronic Supplementary Material 6, Fig. S4).

The posterolateral mandibular surfaces are indented by longitudinal troughs that extend anteriorly from the glenoid articulations (Fig. 5). Similar troughs have been illustrated in PIMUZ A/III0521 (Sachs et al*.*^21^, p. 339, Fig. 2) and *H. tomistomimus* (Benson et al*.*^16^, p. 552, text-Fig. 3), as well as in the rhomaleosaurids *M. victor* (Smith & Vincent^58^, p. 1054, text-Fig. 3C), and *T. wiedenrothi* (Smith & Araújo^60^, p. 105, text-Fig. 7C). Benson and Druckenmiller^1^ considered the lateral mandibular trough to be a synapomorphy for Cretaceous leptocleidians based on its occurrence in *Brancasaurus brancai* (Sachs et al*.*^75^, p. 16, Fig. 4), *Nichollssaura borealis* (Druckenmiller & Russell^76^, p. 7, text-Fig. 5C), and *Plesiopleurodon wellesi* (Benson & Druckenmiller^1^, p. 11, Fig. 4).

The articular forms the posterior margin of the glenoid in MNHNL BU159, which is also situated at the level of the tooth row (Druckenmiller & Russell^74^, p. 45, character 82). The retroarticular processes (Electronic Supplementary Material 6, Fig. S4c) are posteromedially inflected with their mediolateral long axes and posteroventrally oriented with their dorsoventral long axes. The maximum anteroposterior length (115 mm) is equivalent to the length of the glenoid, and the straight dorsal and curving ventral margins conforming to the “Type III” category listed as diagnostic for *P. brachydeirus* and *P. macromerus* by Knutsen (^52^, p. 266, Fig. 6).

#### Dentition

The remains assigned to MNHNL BU159 include three incomplete functional teeth ranging up to 30–36 mm in maximum cross-sectional diameter. The only complete tooth (Fig. 1a) is 79.9 mm in maximum height to the worn apex (28.6 mm from the lingual enamel base), and 15.7 mm in maximum labiolingual diameter. The crown is conical with a sub-circular basal cross-section comparable to the teeth in *Simolestes vorax*^3, 63^, ‘*Pliosaurus*’ *andrewsi*^3,53,63^, *Pachycostasaurus dawni*^63^, *Liopleurodon ferox*^63^, *Brachauchenius lucasi*^48^, *Monquirasaurus boyacensis*^72^, *Kronosaurus queenslandicus*^77^, *Marmornectes candrewi*^12^, *Peloneustes philarchus*^64^, *Cryonectes neustriacus*^18^, *Acostasaurus pavachoquensis*^46^, *Sachicasaurus vitae*^4^, *Megacephalosaurus eulerti*^27^, the multitaxic dental assemblage historically assigned to ‘*Polyptychodon interruptus*’^32^, ‘*Polyptychodon*’ *hudsoni* (D.M. pers. obs.), and numerous other isolated pliosaurid teeth^3,28,30,31,78,79^. The various species of *Pliosaurus*^44,49,52^, *Gallardosaurus iturralde*^80^, *Makhaira rossica*^2^, *Stenorhynchosaurus munozi*^4^, *Luskhan itilensis*^81^, and others^30,37^ alternatively possess trihedral (*M. rossica*, *Pliosaurus* spp.) to sub-trihedral (*G. itturraldei*, *L. itilensis*, *P. kevani*, *S. munozi*), and trihedral-to-trapezoidal^37^ crown shapes. MNHNL BU159 further lacks carinae or cutting edges, although one (e.g. *G. itturraldei*, *L. itilensis*, *P. kevani*), two (most species of *Pliosaurus*), or three (*M. rossica*) prominent carinae may be present in different pliosaurid taxa^2,37,49,80–82^.

As noted by Godefroit^20^, the dental enamel of MNHNL BU159 is densely ornamented by 55 apicobasal ridges that circumscribe the crown base; only 24 enamel ridges extend to the tooth apex with at least eight terminating prior to the worn tip. Some short enamel ridglets are also interspersed between the apicobasal ridges. The enamel ridge cross-sections are sub-triangular, with one branching ridge present on the lingual surface (Godefroit^20^, p. 81). Branching enamel ridges are absent in *S. vorax*^3^ but occur elsewhere in *P. dawni*^63^, *B. lucasi*^48^, *M. eulerti*^5^, ‘*P.*’ *hudsoni* (D.M. pers. obs.), and various isolated pliosaurid teeth^30^ including the specimen identified as the ‘Maryevka pliosaurid’ (SOIKM KP-28988) or Thalassophonea indet. ‘Morphotype 1’ by Zverkov et al.^3^. Notably, while the MNHNL BU159 tooth crown is most compatible with the ‘Maryevka pliosaurid’, it lacks development of the apicobasal ridges as “meandering” cutting edges (Zverkov et al.^3^, p. 829).

#### Postcranial elements

Only a handful of postcranial bones were recovered with MNHNL BU159. Godefroit^20^ listed a cervical rib and some dorsal rib fragments representing the axial skeleton. The cervical rib (Fig. 6a–e) is compact with “co-joined” (Benson & Druckenmiller^1^, appendix 2, character 160) dorsal articular facets separated by a transverse groove that can be traced onto the anterior and posterior surfaces of the rib shaft. Ventrally, the shaft becomes shallowly downcurved and markedly compressed to form lobate anterior and posterior processes (59 mm in combined anteroposterior length) resembling those on the short anterior-most cervical ribs of Jurassic pliosaurids like *Liopleurodon ferox* (Andrews^44^, p. 15, Text-Fig. 4).

The dorsal rib fragments include one reassembled section (Fig. 6g) that has a distinctly circular cross-section with “diamètre de 40 mm” (Godefroit^20^, p. 82). However, we also identified parts of at least three gastral ribs, including two rod-shaped lateral elements with circular cross-sections, and a medial element with distinctively tapered non-bifurcating ends (Fig. 6h).

The appendicular elements of MNHNL BU159 include a disc-like mesopodial that is still encased in matrix (Fig. 6i), and a phalanx (Fig. 6j–m) with constricted shaft and rounded articular ends. Proportionally, this phalanx corresponds to the “long and slender (~ 2–3 times as long proximodistally as broad anteroposteriorly)” state description of Benson & Druckenmiller (^1^, appendix 2, character 270).

**Figure 8:**
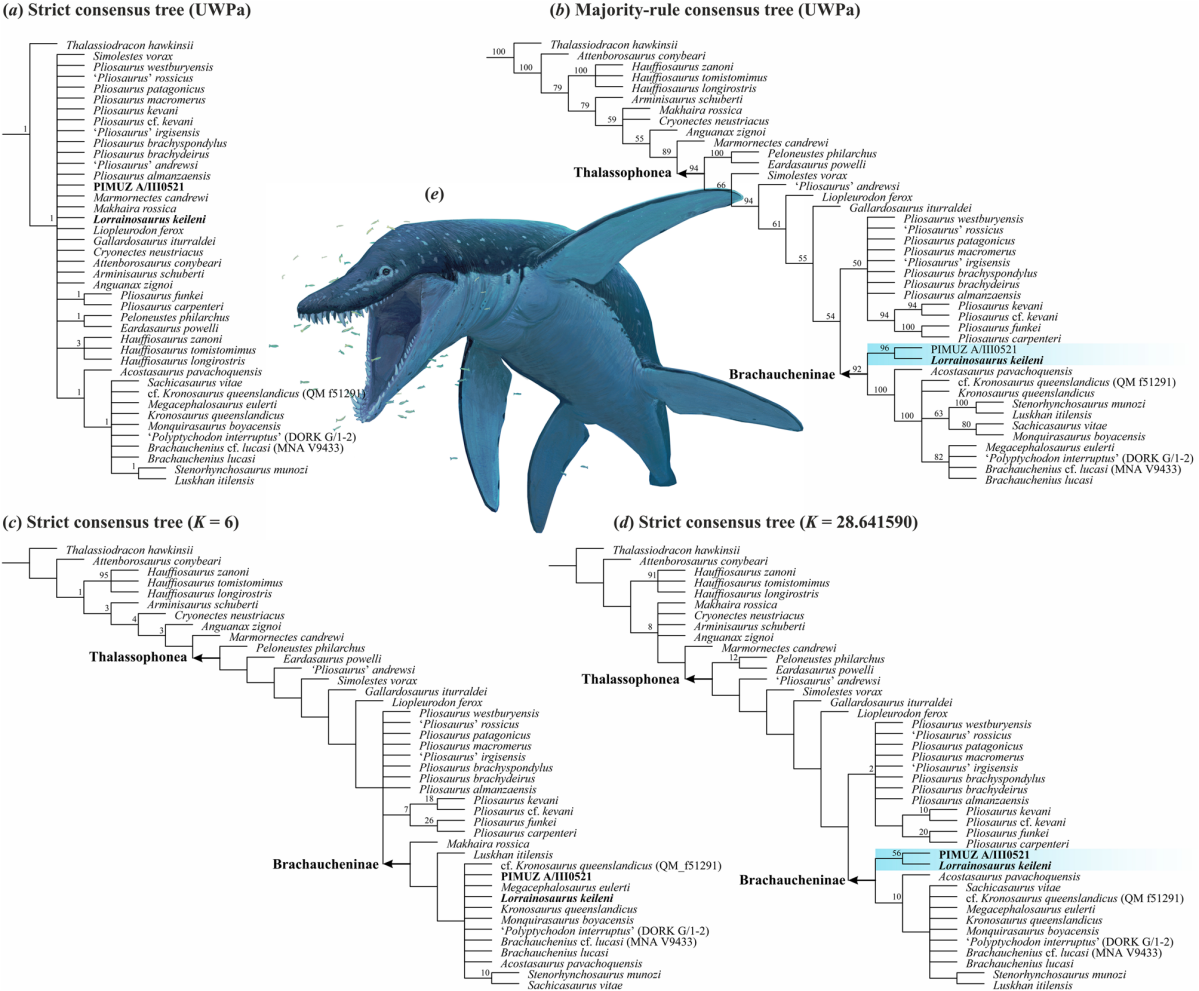
Phylogenetic relationships of *Lorrainosaurus keileni*. (**a**) Strict consensus and (**b**) majority-rule consensus trees from the unweighted parsimony analysis (UPWa); (**c**) strict consensus tree from the weighted parsimony analysis (IWa) with *K* = 6; (**d**) strict consensus tree from IWa with *K* = 28.641590; (**e**) life reconstruction of *Lorrainosaurus keileni* (artwork by Joschua Knüppe); blue shading indicates *L. keileni* and PIMUZ A/III0521. Numbers at nodes indicate (**a**) Bremer support values in UPWa; (**b**) percentage of trees reconstructed in UPWa; (**c**,**d**) symmetric resampling.

Finally, Godefroit^20^ identified a right coracoid (Fig. 7) with maximum length/width of 710/380 mm. This element is highly fractured and missing part of its medial mid-section, but preserves a “plate-like” (Benson & Druckenmiller^1^, appendix 2, character 212) anterior process projecting from the anteromedial edge of the intercoracoid contact. Laterally, the anterior process borders the concave posterior margin of the pectoral fenestra (e.g. as reconstructed in the pectoral girdle of *Simolestes vorax* Andrews^44^, p. 29, Text-Fig. 8). The intercoracoid contact is dorsoventrally thickened and sigmoidal in visceral profile where it supports a mediolaterally directed buttress extending laterally towards the glenoid articulation on the dorsal side. This identifies the element as the left coracoid. Anteriorly and posteriorly, there is a depression adjacent to the buttress. The obverse ventral surface of the coracoid is flat but becomes shallowly concave distally. Posteromedially, the right and left coracoids would have diverged, like those of *Attenborosaurus conybeari* (Sollas^14^ pl. 23, Fig. 3) or *Brachauchenius* cf. *lucasi* (Albright et al*.*^48^, p. 37, Fig. 10), and were more widely separated than in *Peloneustes philarchus* (Andrews^44^, p. 54, Text-Fig. 21), *Simolestes vorax* (Andrews^44^, p. 29, Text-Fig. 8), and *Hauffiosaurus zanoni* (Vincent^17^, p. 347, Fig. 5), but were not embayed as suggested by the rounded posterior extremity. By contrast, the lateral margin of the coracoid is indented by a long concavity. The adjacent glenoid articular facet is offset from the small triangular scapular facet by about 130°. The projecting posterolateral edge (cornu) is slightly wider than the glenoid and rugose, possibly for insertion of the *m. coracobrachialis*^20^.

## Results

### Diagnostic character states of *Lorrainosaurus keileni*

Despite being known from a partial skeleton, the holotype of *Lorrainosaurus keileni* (MNHNL BU159) clearly differs from other currently documented pliosaurid taxa based on a unique combination of characters, including one autapomorphy.

Autapomorphically the splenials in *L. keileni* are transversely broad and ‘wedge-shaped’ and they extend anteriorly to the level of the fourth mandibular alveolus, thus forming a large part of the ventral mandibular symphysis. In other pliosaurids such as *Simolestes vorax*, *Peloneustes philarchus*, and *Liopleurodon ferox*, the splenials are either narrower and/or they do not form a large part of the ventral symphysis (see, e.g. Ketchum & Benson^64^, pl. 3, Fig. 6, Noè^63^, Figs. 42, 139).

The presence of five to six alveoli in the symphyseal part of the mandible was described for *S. vorax*^44^, ‘*Polyptychodon*’ *hudsoni*^45^, and *Acostasaurus pavachoquensis*^46^. *Sachicasaurus vitae* bears three–four tooth positions^4^ whereas other pliosaurids usually have a higher number of teeth adjacent to the symphysis (see description and comparisons above).

A ‘spatulate’ (roughly rosette-shaped) laterally expanded and posteriorly constricted symphyseal mandibular portion was described for the pliosaurids *Simolestes* and *Acostasaurus*^44,46,54^. In other pliosaurids, such as *B. lucasi*, *P. philarchus*, and *L. ferox*, the symphyseal portion is usually more elongate and less transversely expanded (see e.g. Albright et al*.*^48^, Fig. 3B, Andrew^44^, plate 2, Fig. 1, Ketchum & Benson^64^, plate 3, Fig. 6).

The presence of a lateral trough anterior to the mandibular glenoid is a character that distinguishes *Lorrainosaurus* from all other pliosaurids except for *Hauffiosaurus* and *P. kevani* (see Benson & Druckenmiller^1^, character 121).

The retroarticular process in *Lorrainosaurus* is shorter than the glenoid fossa. A similar condition of a retroarticular process that is either shorter or subequal in length with the mandibular glenoid was described for a number of pliosaurids including *Pliosaurus* spp., *L. ferox*, *Megacephalosaurus eulerti* or *B. lucasi* (see Benson & Druckenmiller^1^, character 116). However, some taxa such as *S. vorax* (see Noè^63^, Fig. 125), *Arminisaurus schuberti*^19^, *Cryonectes neustriacus*^18^, *Hauffiosaurus* spp.^16^ or *A. pavachoquensis*^46^ have a retroarticular process that is longer than the glenoid fossa.

The retroarticular process in *Lorrainosaurus* has also a dorsoventral long axis that is posteroventrally inclined and a transverse long axis that is slightly posteromedially inflected. Such morphology also occurs in *Rhaeticosaurus mertensi*^83^, *A. schuberti*^19^, *Kronosaurus queenslandicus*^51^, *S. vitae*^4^, and *A. pavachoquensis* ^46^, but is absent in other pliosaurids (see Benson & Druckenmiller^1^, characters 122 and 123).

Finally, the posteromedial side of the coracoid of *L. keileni* is strongly divergent and curves laterally. A similar condition is present in the pliosaurids *Attenborosaurus conybeari*^14^ and *Brachauchenius* cf. *lucasi*^48^, whereas in other pliosaurids such as *S. vorax*, *Hauffiosaurus zanoni*, and *Luskhan itilensis* the posterior portions of the coracoids are only slightly split^6,17,44^ (Electronic Supplementary Material 6, Fig. S5).

### Phylogenetic relationships

Although the tree topology resulting from UPWa is unresolved (Fig. 8a), the majority-rule consensus tree and IWa trees find topologies broadly congruent with those inferred through other recent studies assessing the phylogenetic relationships of pliosaurid plesiosaurs (e.g. ^6,11,34,84^), and reconstruct *L. keileni* and PIMUZ A/III0521 within Thalassophonea, as earliest-diverging members of Brachaucheninae. Owing to the insufficient completeness of *L. keileni* and PIMUZ A/III0521, such placement needs to be treated with caution. Rather, it should only be considered to support the thalassophonean origin for the grouping.

*Lorrainosaurus keileni* + PIMUZ A/III0521 are returned as sister taxa in a subset of trees using an IWa with *K* = 6, based on their posteroventrally inflected dorsoventral orientation of the long axis of the retroarticular process (122:0 → 1). Alternatively, *L. keileni* + PIMUZ A/III0521 are united by the possession of a medially bowed mandible anterior to the glenoid (111: 1 → 0), and a prominent longitudinal trough incising the lateral surface of the mandible anterior to the glenoid (121:0 → 1) under IWa settings with *K* = 28.641590 (63 steps of homoplasy downweighted 10 times). *Lorrainosaurus keileni* + PIMUZ A/III0521 additionally share a posteromedially inflected mediolateral orientation of the long axis of the retroarticular process (123:0 → 1) with brachauchenines.

Finally, enforced monophyly of *L. keileni* + *Simolestes vorax* using a UPWa with identical settings increased both the number of most parsimonious trees (MPTs) and length (L): ‘New Technology’ search results yielding 55 MPTs, L = 2056 versus 34 MPTs, L = 2051 without enforced monophyly; ‘Traditional’ searches using trees saved to RAM all reached 200,000 MPTs.

### Multivariate analyses

**Figure 9:**
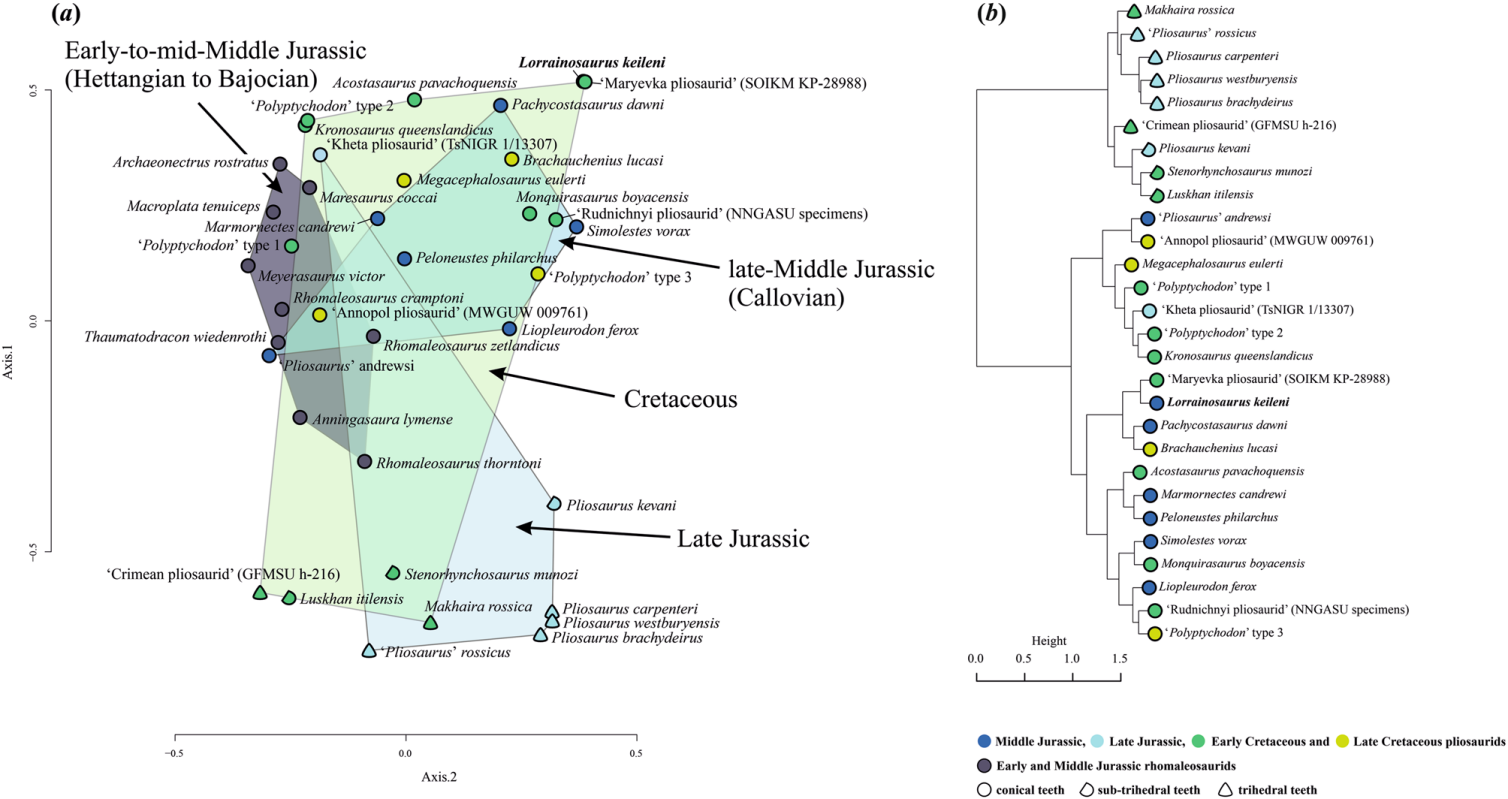
Dental morphospace occupation of *Lorrainosaurus keileni*. (**a**) Plot of principle coordinates analysis (PCoA) results with the PCo1/PCo2 axes showing *L. keileni* (bold type) versus rhomaleosaurid (grey shading) and Jurassic (blue shading) to Cretaceous (green shading) thalassophonean pliosaurids. (**b**) Cluster dendrogram of conical (circles), sub-trihedral (semicircles) and trihedral (triangles) thalassophonean tooth crown morphologies from the Middle (dark blue) and Late Jurassic (light blue) to Early (dark green) and Late Cretaceous (light green).

Dental morphology has been used to reconstruct the ecomorphological affinities of pliosaurids^3^ and evaluate the similarities between their teeth^5,31^. Consequently, to assess the morphospace occupation of *Lorrainosaurus keileni* (MNHNL BU159), we augmented a published dataset of character state scores derived from the teeth of Jurassic and Cretaceous pliosaurids^3,5,31^, combined with novel scores for Early and Middle Jurassic rhomaleosaurids. Plots generated from a principal coordinates analysis (PCoA) and cluster analysis (CA) in *R*^38^ clearly segregated *L. keileni* as an extreme positive outlier compared to rhomaleosaurids, which are broadly distributed along PCo1 but on a negative side of PCo2 (Fig. 9a, Electronic Supplementary Material 1, Fig. S2). *Lorrainosaurus keileni* is also distinct from later Jurassic pliosaurids along PCo1, although *Simolestes vorax* and the Callovian taxon *Pachycostasaurus dawni*^85^ are closely situated on PCo2, and PCo1/PCo3, respectively (Electronic Supplementary Material 1, Figs S2 and S3). Finally, *L. keileni* shares morphospace occupation with the ‘Maryevka pliosaurid’, which represents an indeterminate thalassophonean^3^. This result is mirrored by our CA dendrogram (Fig. 9b), which groups *L. keileni* and the ‘Maryevka pliosaurid’ with *P. dawni* and *B. lucasi* amongst pliosaurids sharing conical tooth crowns, as opposed to those with sub-trihedral or trihedral-shaped teeth epitomised by species of the Late Jurassic taxon *Pliosaurus*^43,49^.

## Discussion and conclusions

At 1.33 m and  ~ 1.5 m in maximum length, the mandibles of *Lorrainosaurus keileni* and PIMUZ A/III0521, respectively, proportionately exceed that of *Simolestes vorax* at 970 mm^44^ and approach the mandibular lengths of later-diverging thalassophonean taxa, such as the Cretaceous brachauchenines *Brachauchenius lucasi* at 1.25 m^48^ or *Megacephalosaurus eulerti* at 1.71 m^50^. This suggests that large skulls appeared early in their evolutionary history (at least by the early Bajocian^21^, after ~ 171 Ma). By contrast, mandibular lengths of even the largest rhomaleosaurids are less spectacular, with the Early Jurassic *Rhomaleosaurus zetlandicus* at 695 mm^86^, *Atychodracon megacephalus* at 830 mm^59^, the Middle Jurassic *Borealonectes russelli* at up to 512 mm^87^, and *Maresaurus coccai* at a maximum of ~ 1.1 m^21^. Presumably, such variation not only reflected smaller skull sizes but also different feeding styles and ecologies. Certainly, while the jaws of gigantic pliosaurids were seemingly capable of massive adductive forces, their characteristically elongate and structurally ‘weak’ skull shape was hydrodynamically optimised for fast crushing bites^88^. Rhomaleosaurids, in turn, had broader heads and apparently employed vigorous shake and twist feeding to dismember prey^89^. These interpretations are consistent with their dental morphologies, which as we show, diverged from curved and pointed teeth in rhomaleosaurids, possibly adapted for feeding on fish and cephalopods^90^, to robust conical teeth in early thalassophoneans, like *L. keileni* that may have fed on fleshy prey including larger fish and aquatic tetrapods^3,90^. Interestingly, our results further suggest that some pliosaurids dentally converged on rhomaleosaurids concurrent with their decline across the later-Middle Jurassic (Callovian, up to ~ 161.5 Ma)^91^, while others specialised towards trihedral cutting teeth^3^ by the Late Jurassic (before the Kimmeridgian, ~ 154.8 Ma). Lastly, the archetypal conical-toothed morphology typified by *L. keileni* subsequently dominated in later-Early Cretaceous thalassophoneans (from the Aptian, ~ 121.4 Ma)^3^.

The incipient radiation of macropredatory pliosaurids has been associated with a landmark turnover of Early-to-Middle Jurassic marine reptile assemblages inhabiting the northwestern Tethyan epicontinental periphery of what is today western Europe^22^. Notably, this coincides with abrupt oceanic cooling over the earliest Middle Jurassic interval (Aalenian, ~ 174.7 Ma)^92^, and accompanying extinctions affecting nektonic invertebrates, in particular cephalopods^93,94^. The ensuing recovery of major belemnite (Belemnopseina)^93^ and ammonite (Ammonitina)^94^ groups was distinguished by biogeographical provincialism, involving separation into distinct Tethyan and Boreal faunas^93,95^.

The adaptive diversification of thalassophoneans as apex-predators might have facilitated via local food chain disruptions triggering ecological niche vacation by rhomaleosaurids and other larger-bodied marine reptiles in the northwestern Tethys^22,96^. However, the consequent innovation and global dispersal of macropredatory pliosaurids patently did not accelerate until the early-Late Jurassic (Oxfordian, by ~ 154.8 Ma)^97^, and was approximately concurrent with the final extinction of rhomaleosaurids (potentially induced by climatic warming across the Middle-to-Late Jurassic transition^91^), as well as tectonic fragmentation that established seaway connections permitting migration between the northwestern Tethys and Palaeo-Pacific^98^.

In summary, our results demonstrate that thalassophonean pliosaurids were the geologically longest-ranging clade of macropredatory marine tetrapods with a fossil record spanning  ~ 80 Ma. Their advent paralleled a regional marine faunal turnover in the earliest Middle Jurassic^22,93,94^ that was perhaps associated with rapid oceanic temperature changes and the progressive decline of coeval macrophagous marine reptiles specialised for feeding on cephalopods^97^. These included coeval large-bodied rhomaleosaurids^90^, which persisted until the latest Middle Jurassic^91^ but were likely not direct competitors. Rather, rhomalosaurids were ecomorphologically partitioned from the earliest thalassophoneans, which otherwise pioneered plesiosaur macropredator niches to dominate the Mesozoic seas.

### Supplementary Information

Supplementary Information 1.

Supplementary Information 2.

Supplementary Information 3.

Supplementary Information 4.

Supplementary Information 5.

Supplementary Figures.

## Data Availability

The data are provided in the Electronic Supplementary Material.
