# Supplementary material for: The rise of macropredatory pliosaurids near the Early-Middle Jurassic transition
Source: Sci Rep. 2023 Oct 16;13:17558. doi: 10.1038/s41598-023-43015-y (PMC10579310; doi:10.1038/s41598-023-43015-y)
Supplement: Supplementary file 1 — Supplementary Information 1. [file 41598_2023_43015_MOESM1_ESM.docx]

Electronic Supplementary Material 1 for:

**The rise of macropredatory pliosaurids near the Early-Middle Jurassic transition**

Sven Sachs, Daniel Madzia, Ben Thuy and Benjamin P. Kear


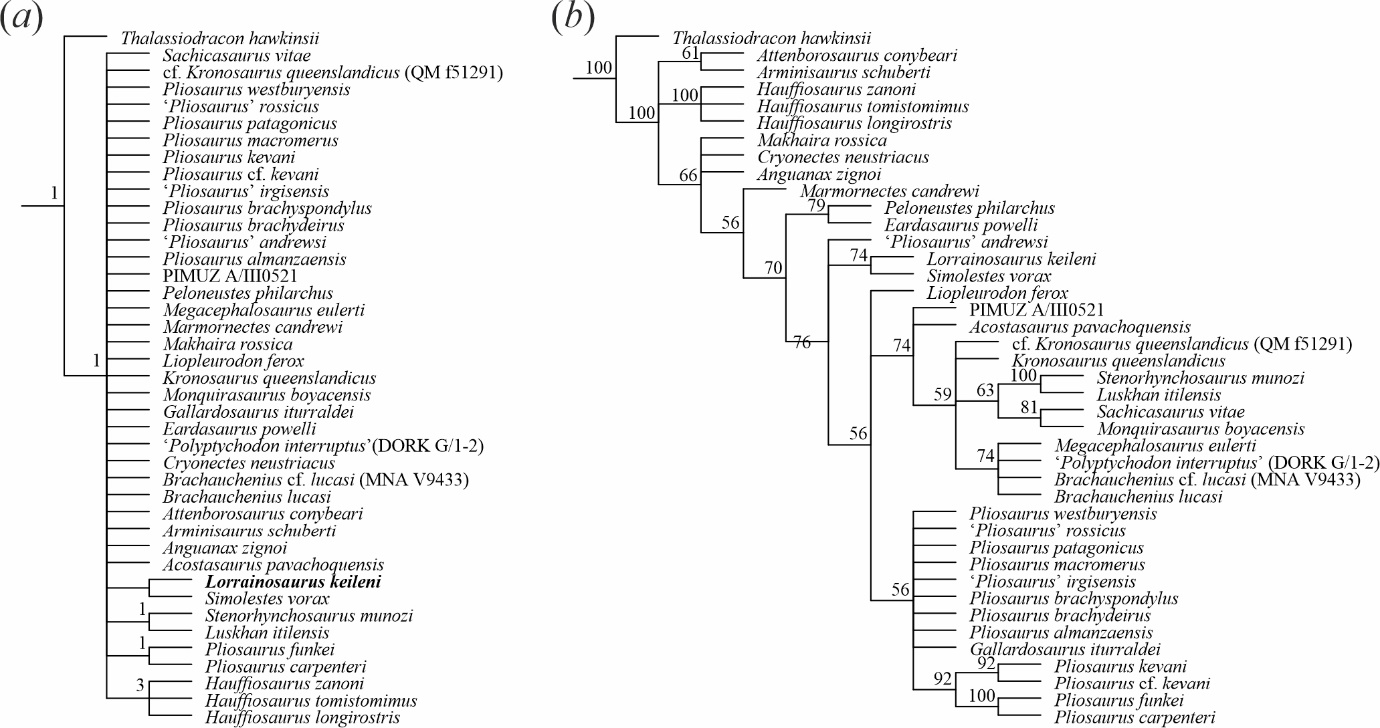


**Figure S1**. Results of the constrained unweighted parsimony analysis showing (*a*) the strict consensus tree and (*b*) the majority-rule consensus tree. Values on nodes on (*a*) indicate the Bremer support, while those on nodes on (*b*) show the percentage of trees reconstructed in the analysis.


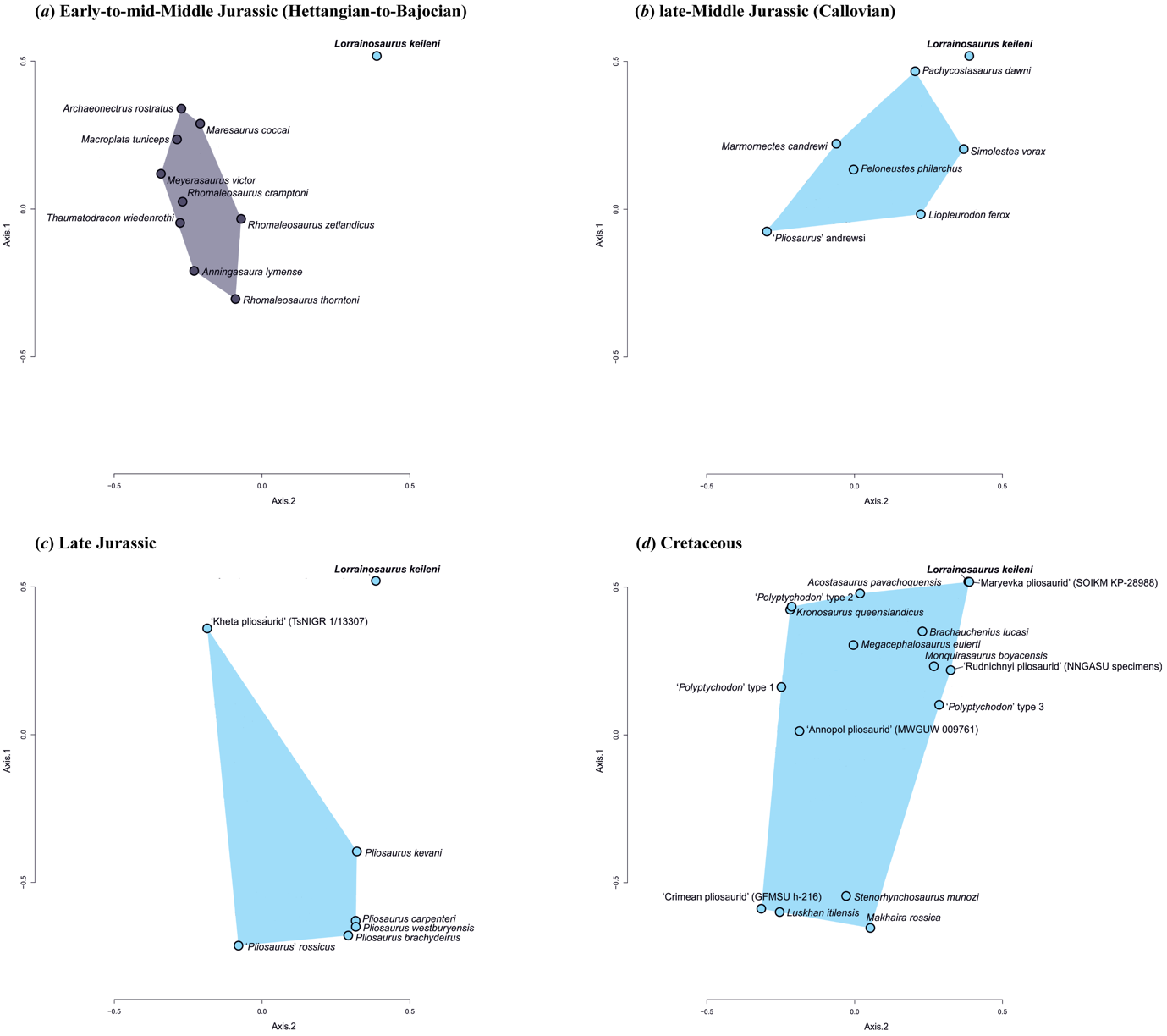


**Figure S2**. Principal Coordinates Analysis (PCoA) plots (PCo1/PCo2) showing dental morphospace occupation of *Lorrainosaurus keileni* (bold type) versus various thalassophonean pliosaurid (blue circles) and rhomaleosaurid (grey circles) taxa from the (*a*) Early-to-mid-Middle Jurassic (Hettangian-to-Bajocian), (*b*) late-Middle Jurassic (Callovian), (*c*) Late Jurassic, (*d*) Cretaceous.


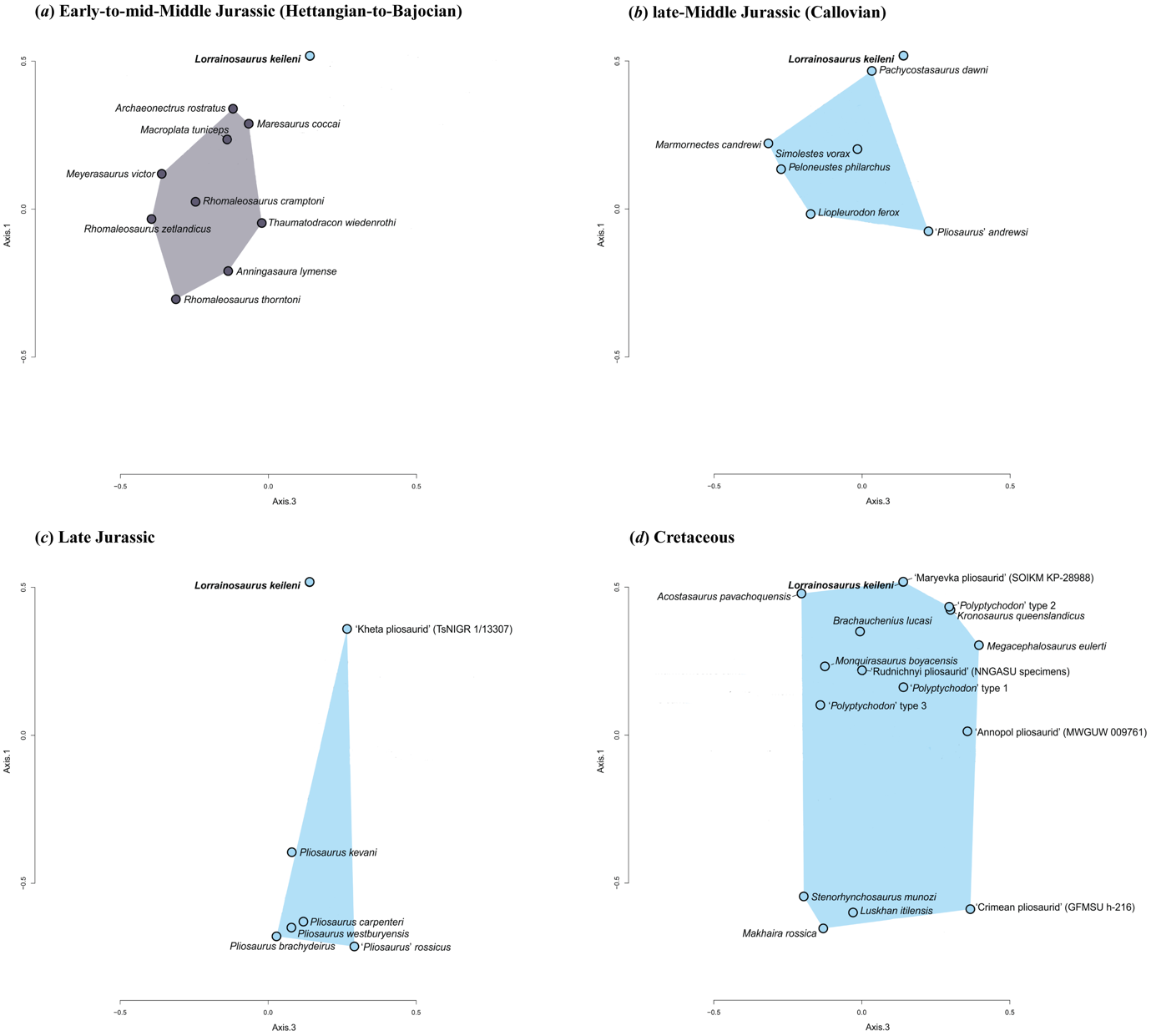


**Figure S3**. PCoA plots (PCo1/PCo3) showing dental morphospace occupation of *Lorrainosaurus keileni* (bold type) versus various thalassophonean pliosaurid (blue circles) and rhomaleosaurid (grey circles) taxa from the (*a*) Early-to-mid-Middle Jurassic (Hettangian-to-Bajocian), (*b*) late-Middle Jurassic (Callovian), (*c*) Late Jurassic, (*d*) Cretaceous.

**Table S1.** Summary of numerical results from the phylogenetic analyses. Abbreviations: BS, best score; CI, Consistency Index; MPT, most parsimonious trees; ‘NT’, ‘New Technology’ search; RI, Retention Index; ‘TS’, ‘Traditional search’.

| **Run** | **MPT (‘NT’)** | **BS** | **MPT (‘TS’)** | **CI** | **RI** |
| --- | --- | --- | --- | --- | --- |
| **Unweighted parsimony (unconstrained)** | 34 | 2051 | 200,000 | 0.195 | 0.688 |
| **Weighted parsimony (*K* = 6)** | 235 | 135.36365 | 200,000 | 0.193 | 0.684 |
| **Weighted parsimony (*K* = 28.641590)** | 142 | 46.74397 | 35,190 | 0.194 | 0.687 |
| **Unweighted parsimony (constrained)** | 55 | 2056 | 200,000 | 0.194 | 0.687 |
