# Supplementary material for: The rise of macropredatory pliosaurids near the Early-Middle Jurassic transition
Source: Sci Rep. 2023 Oct 16;13:17558. doi: 10.1038/s41598-023-43015-y (PMC10579310; doi:10.1038/s41598-023-43015-y)
Supplement: Supplementary file 2 — Supplementary Information 2. [file 41598_2023_43015_MOESM2_ESM.docx]

Electronic Supplementary Material 2 for:

**The rise of macropredatory pliosaurids near the Early-Middle Jurassic transition**

Sven Sachs, Daniel Madzia, Ben Thuy and Benjamin P. Kear

1. Data for the phylogenetic analyses of Plesiosauria

(a) Character list

**1.** **Transverse constriction of the rostrum at the premaxilla-maxilla suture:** absent (0); present (1).

**2.** **Maxilla, lateral expansion of maxilla posterior to maxilla-premaxilla suture accommodates expanded caniniform bases [‘roots’]:** absent (0); present (1).

**3.** **Ratio of orbit length in dorsal view to temporal fenestra length:** 0.3–0.7 (0); >0.8 (1).

**4.** **Ratio of pre-orbital skull length to total skull length measured in dorsal view:** <0.44 (0); 0.45–0.55 (1); >0.56 (2).

**5.** **Orbit, ventral margin of outline in lateral view:** concave, resulting in a suboval orbital outline (0); convex, resulting in reniform orbital outline with prominent lobate anterior extension (1).

**6.** **Dorsal margin of orbit, outline in dorsolateral view:** concave, forming part of a suboval orbit (0); convex, skull roof projects into orbit (1).

**7.** **Relative skull length compared to length of dorsal series:** 0.20–0.30 (0); 0.31–0.39 (1); >0.40 (2).

**8.** **Inclination of the suspensorium:** sub-vertical or weakly inclined (~80–90º) (0); significantly inclined (<70º) (1).

**9.** **Relative positions of external and internal nares:** internal naris posterior to external naris (0); nares overlap (1); internal naris anterior to external naris (2).

**10.** **Position of the mandibular glenoid fossa:** coplanar with the occipital condyle (0); just posterior to the occipital condyle (1); far posterior to occipital condyle, distance at least equal to basicranial (basioccipital+basisphenoid) length (2).

**11.** **Fluted ornamentation of bone surface around the dorsal orbit margin (on prefrontal or postfrontal):** absent (0); striations oblique to orbit margin (1); striations perpendicular to orbit margin (2).

**12.** **Temporal bar, suborbital margin:** smoothly curved (0); squared-off posteroventral margin of suborbital skull forms abrupt edge (1).

**13.** **Alveolar margin of upper jaw in lateral view:** approximately straight or weakly convex (0); undulating, forming 'scalloped' margin (1).

**14.** **Premaxilla, external surfaces:** marked neurovascular foramina but otherwise smooth (0); numerous sharp rugose crests (1); consistently undulating with rounded ridges (2).

**15.** **Premaxilla contact along the dorsal midline:** contacts anterior extension of frontals only (0); partially overlaps the frontal along the midline (1); overlaps the entire length of the frontal along the dorsal midline and contacts the parietal (2).

**16.** **Premaxilla, posterior termination:** tapering and non-interdigitating or weakly interdigitating (0); broad, deeply interdigitating suture with the frontal or parietal (1).

**17.** **Premaxilla, dorsomedian ridge:** absent or indistinct (0); prominent, forming either a narrow crest, a broad bar-like ridge, or a mound-like eminence on the dorsomedian surface of the rostrum (1).

**18.** **Premaxilla, morphology of dorsomedian ridge:** narrow and crest-like (taller than wide) (0); broad, occupying most of the internarial width of the rostrum (1); posterior mound (2).

**19.** **Premaxilla, dorsomedian ridge location:** anterior (0); posterior (1); elongate, extends from interorbital region to rostral tip (2).

**20.** **Premaxilla dorsomedian foramen:** absent (0); present (1).

**21.** **Premaxilla, participation in medial rim of external naris:** participates broadly along anteroposterior length of external naris (0); does not participate (1); small contact at anterodorsal corner of external naris (2).

**22.** **Premaxilla, constriction of posteromedian process at level of external naris:** absent (0); present, and does not expand to original width posterior to naris (1); present, but premaxilla expands to original width posterior to naris (2).

**23.** **Premaxilla-maxilla sutures:** converging posteromedially gradually, for entire length (0); anterior portion extends dorsomedially then abruptly curves posteriorly, resulting in a parallel-sided appearance of the posterior process of the premaxilla (1).

**24.** **Premaxilla-maxilla sutures, morphology anteriorly:** curved, but only weakly interdigitating, sinuous, or straight (0); pronounced, anteroposteriorly interdigitating contact with ‘zig-zag’ appearance (1).

**25.** **Maxilla and dentary, posterior extent of maxillary tooth row:** around orbital midlength or more anteriorly (0); ventral to postorbital bar (1); ventral to temporal fenestra midlength (2).

**26.** **Maxilla-squamosal contact:** absent (0); present (1).

**27.** **Maxilla participation in internal naris:** participates (0); does not participate (1).

**28.** **Maxilla, trough or depressed region anterior to external naris:** absent (0); present, defined laterally by a longitudinal ridge but does not extend far anteriorly (1); prominent longitudinal trough extends most of the prenarial length of the maxilla (2).

**29.** **Posteromedial extension of the maxilla:** extends to anteromedial margin of the external naris (0); extends to midpoint of the medial margin of the external naris (1); extends posteromedial to the external naris (2).

**30.** **Maxilla, posteromedial (=posterodorsal) portion:** not subdivided, forms simple sheet of bone (0); subdivided by anteroposteriorly oriented fissures (1).

**31.** **Frontal participation in rim of external naris:** does not participate (0); participates (1).

**32.** **Frontal, posterolateral process:** present (0); absent (1); inapplicable, premaxilla contacts parietal (?).

**33.** **Frontal participation in orbital margin:** participates (0); does not participate, excluded by prefrontal-postfrontal contact (1).

**34.** **Lacrimal:** absent, maxilla participates in orbit margin (0); present, maxilla excluded from orbit margin (1).

**35.** **Prefrontal participation in rim of external naris:** does not participate (0); participates (1).

**36.** **Postfrontal participation in orbital margin:** participates (0); does not participate, excluded by postorbital-frontal contact (1).

**37.** **Jugal participation in orbital margin:** participates (0); does not participate, excluded by maxilla-postorbital contact (1).

**38.** **Jugal, size and anteroposterior length:** large, with horizontal long axis, extends anteriorly at least one-third of orbital length (0); short, terminates around posterior orbital margin (1); very reduced and anteroposteriorly short with vertical long axis (2).

**39.** **Jugal, shape of anterior margin:** tapering, embayed by orbit margin, or contacts ‘lacrimal’ (0); squared off (1).

**40.** **Jugal-squamosal contact:** absent (0); present (1).

**41.** **Jugal-squamosal contact morphology:** subvertical and interdigitating (0); subhorizontal for most of length, not interdigitating (1); inapplicable, contact absent (?).

**42.** **Postorbital-squamosal contact:** present, excluding jugal from the margin of the supratemporal fenestra (0); absent, and jugal enters margin of the temporal fenestra (1).

**43.** **Postorbital posterolateral process length:** long, extending posteriorly for at least two-thirds of the temporal fenestra length (0); prominent, but not elongate, extending approximately one-third of temporal fenestra length (1); short or absent (2).

**44.** **Pineal foramen:** present (0); absent (1).

**45.** **Pineal foramen, surrounding elements:** enclosed entirely within the parietals (0); contacts the frontals or premaxillae anteriorly (1); inapplicable, pineal foramen absent (?).

**46.** **Pineal foramen-location relative to postorbital bar:** level with postorbital bar (0); just posterior to postorbital bar (1).

**47.** **Morphology of pineal foramen:** suboval (0); anteroposteriorly elongate and slot-like (1); inapplicable, pineal foramen absent (?).

**48.** **Inter-squamosal suture along the dorsal midline in lateral view:** low and rounded (0); raised ~1/3 orbit height dorsally relative to skull table (1); raised abruptly and substantially dorsally relative to skull table (2).

**49.** **Parietal vault in dorsal view:** mediolaterally narrow, lateral surfaces weakly concave or slightly sinuous (0); expanded to approximately one-third the mediolateral width of the skull, lateral surfaces convex, forming abrupt ‘lateral angle’ of Smith & Dyke (2008) (1); strongly expanded, equal to at least half the transverse width of the posterior cranium, lateral surfaces concave (2).

**50.** **Parietal, sagittal crest height:** crest absent, dorsal surface of parietal broad and flat (0); low, transversely convex (1); high, transversely compressed sheet (2); very high, forming convex dome in lateral view rising above the skull table (3).

**51.** **Parietal ornamentation adjacent to the pineal foramen:** ornamentation absent (0); ornamented with numerous anteroposteriorly oriented ridges that extend from the pineal foramen, surface flat or slightly concave along midline (1); parietal with raised midline ridge (2); ‘parietal table’ [triangular depression between pineal foramen and sagittal crest; Druckenmiller & Russell, 2008*b*] present (3).

**52.** **Parietal, anterior extension:** short or absent, parietal extends to the level of the temporal bar (0); long, parietal extends to orbital midlength or more anteriorly (1); very long, parietal extends to anterior orbit margin or more anteriorly (2).

**53.** **Squamosal arch, posterior margin in dorsal view:** dorsal processes extend anterolaterally (0); approximately straight, squamosal dorsal processes extend laterally from midline contact (1); V-shaped, squamosal dorsal processes extend posterolaterally (2).

**54.** **Squamosal arch, cross section of dorsal process of squamosal:** dorsoventral/mediolateral width subequal to or less than anteroposterior width (0); anteroposteriorly compressed (1).

**55.** **Temporal emargination:** moderately embayed, temporal bar arches dorsal to a horizontal line drawn through the tooth row (0); weakly embayed, or not embayed, temporal bar does not significantly arch dorsally (1).

**56.** **Temporal bar, dorsoventral thickness:** low, significantly less than height of orbit (0); high, subequal to 2/3 or greater than height of orbit (1).

**57.** **Squamosal, anterior extent:** ventral to postorbital bar (0); significantly posterior to postorbital bar (1).

**58.** **Inter-squamosal suture along the posterodorsal midline:** flat (0); prominent, ‘bulb-like’ posterior extension (1); low, mediolaterally broad posterior convexity in dorsal view (2).

**59.** **Squamosal-quadrate foramen:** absent (0); present (1).

**60.** **Squamosal-quadrate contact, length of ventromedial process of the squamosal:** short, approximately half the dorsoventral length of the quadrate shaft or less (0); long, extends further ventrally than half the quadrate shaft length (1).

**61.** **Squamosal, outline of posterior margin in lateral view:** approximately straight (0); dorsal portion inflected abruptly anterodorsally (1).

**62.** **Position of tooth row in lateral view:** collinear with the mandibular glenoid fossa (0); considerably higher than the glenoid fossa (1).

**63.** **Notochordal pit on occipital condyle:** absent (0); present (1); occipital condyle scored by multiple pits and deep grooves (2).

**64.** **Notochordal pit on occipital condyle, location:** centrally or at least partly within ventral two-thirds of condyle (0); comfortably within dorsal one-quarter of condyle (1); inapplicable, notochordal pit absent (?).

**65.** **Occipital condyle constriction:** complete, exoccipital facets are separated from the occipital condyle by a groove (0); incomplete because exoccipital facets contact the occipital condyle (1); or constricting groove altogether absent, even ventrally (2).

**66.** **Ventral process of the basioccipital:** absent, weakly developed or wide, flat, relatively smooth, with a thin plate present [small ‘step’ between condyle and ventral surface of basioccipital] (0); very prominent, ventrally projecting plate present (1).

**67.** **Foramen magnum, proportion of foramen enclosed by supraoccipital:** less than one-third (0); approximately half (1).

**68.** **Exoccipital-opisthotic body, dorsoventral height:anteroposterior width ratio:** <1.1 (0); 1.2–1.3 (1); >1.35 (2).

**69.** **Exoccipital, foramina in lateral surface:** one (0); two (1); three/four (2).

**70.** **Opisthotic, paraoccipital process length relative to height of exoccipital body:** subequal (0); long, at least 1.3 times as long as body height (1).

**71.** **Opisthotic, orientation of paraoccipital process relative to ventral surface of exoccipital in posterior view:** inclined dorsally (0); paraoccipital process oriented parallel to ventral surface of exoccipital (1); inclined ventrally (2).

**72.** **Opisthotic, morphology of articulation with suspensorium:** anterior surface of expanded lateral end makes broad contact with suspensorium (0); lateral end unexpanded, lateral/terminal surface makes narrow contact with suspensorium (1).

**73.** **Opisthotic, shaft of paraoccipital process cross section:** subcircular, dorsoventral height subequal to anteroposterior width (0); dorsoventrally flattened; anteroposterior width much greater than dorsoventral height (1).

**74.** **Opisthotic, shaft curvature seen in posteromedial view:** absent, shaft approximately straight (0); curves dorsodistally (1).

**75.** **Prootic, anteroventral process:** long, meaning that ventral anteroposterior length is much greater than dorsal anteroposterior length (0); short, dorsal anteroposterior length is slightly greater than ventral (1).

**76.** **Supraoccipital morphology in lateral view:** wider than tall (0); or taller than wide (1).

**77.** **Posteromedian ridge of supraoccipital:** present (0); absent (1).

**78.** **Posteromedian process of supraoccipital:** present (0); absent (1).

**79.** **Supraoccipital, minimum mediolateral width of exoccipital rami in posterior view:** a single ramus is substantially narrower than the foramen magnum (0); subequal to foramen magnum (1).

**80.** **Basisphenoid (or parabasisphenoid) contribution to the basioccipital tuberosities:** contributes, enclosing posterior half of tuber and forming part of the articular surface for the pterygoids (0); does not contribute (1).

**81.** **Deep notch in the posterior margin of the body of the basisphenoid [‘clivus’]:** absent (0); present (1).

**82.** **Basisphenoid-basioccipital connection in ventral view:** fontanelle absent (0); fontanelle present (1).

**83.** **Parasphenoid (or parabasisphenoid), morphology of ventral surface within interpterygoid vacuity:** mediolaterally concave (0); flat or weakly convex (1); bears distinct midline keel (2); inapplicable, parasphenoid does not extend far into posterior interpterygoid vacuity (?).

**84.** **Parasphenoid, posterior extent on midline:** terminates within anterior one-third of interpterygoid vacuity or more anteriorly (0); terminates just anterior to basioccipital-basisphenoid contact on ventral surface of basicranium (1); ventrally underlaps basioccipital so basisphenoid-basioccipital contact is not visible in ventral view (2); as state ‘2’ but also underlaps pterygoids ventrally (3).

**85.** **Parasphenoid, cultriform process length:** extremely short, effectively absent (0); present forming prominent anterior projection (1).

**86.** **Parasphenoid, ventral surface anteriorly:** covered by pterygoids anterior to the posterior interpterygoid vacuities (0); visible through V-shaped notch in posterior pterygoid contact anterior to posterior interpterygoid vacuities (1).

**87.** **Suborbital fenestra bordered by ectopterygoid and maxilla:** absent (0); present (1).

**88.** **Lateral palatal fenestration bordered by palatine and pterygoid:** absent (0); present (1).

**89.** **Element demarcating the anterior margin of the subtemporal fenestra:** the ectopterygoid and pterygoid together (0); exclusively the ectopterygoid (1).

**90.** **Palate, foramina between maxilla and vomer anterior to internal naris:** absent (0); present (1).

**91.** **Posterior extent of the vomers:** extend to the internal nares (0); extend posterior to the internal nares (1).

**92.** **Pterygoid-vomer contact:** pterygoid does not separate vomers along midline (0); pterygoid separates vomers along the midline posteriorly (1).

**93.** **Palatine, participation in the rim of the internal naris seen in ventral view:** participates (0); does not participate (1).

**94.** **Palatines, median contact:** do not contact (0); contact (1).

**95**. **Pterygoid, anterior termination:** tapering (0); transversely broad and interdigitates with vomer (1).

**96.** **Pterygoids, anterior interpterygoid vacuity:** absent (0); present (1).

**97.** **Pterygoids, anterior interpterygoid vacuity, mediolateral width:** narrow, approximately one-fifth of combined pterygoid width at vacuity midlength (0); broad, at least one-third of combined pterygoid width at vacuity midlength (1); inapplicable, vacuity absent (?).

**98.** **Pterygoids, posterior contact with basicranium:** loose, overlapping contact (0); firm sutural contact, ventral surface of pterygoids level with ventral surface of basioccipital (1); narrow anteromedial process of the posterior pterygoid contacts parabasisphenoid primarily or only, forming a butt joint (2).

**99.** **Pterygoids, midline contact posterior to posterior interpterygoid vacuity:** absent (0); present posteriorly, but very small (1); present, pterygoid contact for more than two-thirds of their anteroposterior length posterior to posterior interpterygoid vacuity (2).

**100. Pterygoid lateral to the posterior interpterygoid vacuities:** flat (0); forms ventrolaterally directed flange with long axis oriented posteromedially (1); relatively broad mediolaterally and forms anteroposteriorly oriented trough or dished, with a marked central depression (2).

**101.** **Pterygoid flange, posterior midline contact:** flanges do not contact on midline posterior to posterior interpterygoid vacuity (0); flanges contact on midline, enclosing semicircular fossa posterior to posterior interpterygoid vacuity (1); inapplicable, pterygoid flanges absent (?).

**102.** **Pterygoid, posterolateral portion of pterygoid:** does not form squared lappet (0); forms squared lappet that distinctly underlaps quadrate ramus of pterygoid (1).

**103.** **Posterior interpterygoid vacuities, ratio of maximum length to combined width:** <1.2 (0); 1.3–1.6 (1); 1.8–2.5 (2); >2.6 (3).

**104.** **Posterior interpterygoid vacuities, location of midpoint relative to anterior margin of subtemporal fossa:** posterior to anterior margin of fossa (0); approximately level with anterior margin of fossa or more anterior (1).

**105.** **Basioccipital body, exposure posterior to pterygoid midline contact:** absent, pterygoids cover ventral surface of basioccipital anterior to condyle (0); present, semioval portion of basioccipital exposed between pterygoids anterior to condyle (1).

**106.** **Posterior border of anterior interpterygoid vacuity:** bordered by pterygoid (0); bordered by parasphenoid (1); inapplicable, anterior interpterygoid vacuity absent (?).

**107.** **Anterior border of anterior interpterygoid vacuity:** bordered by pterygoid (0); bordered by vomer (1); inapplicable, anterior interpterygoid vacuity absent (?).

**108.** **Morphology of the posterior border of the anterior interpterygoid vacuity:** concave/rounded (0); parasphenoid projects into anterior interpterygoid vacuity (1); inapplicable, parasphenoid does not contact anterior interpterygoid vacuity, which is thus enclosed posteriorly by the pterygoids (?).

**109.** **Ectopterygoid/pterygoid boss/flange:** absent (0); ventrally deflected posterior margin forms flange (1); rugose ventral boss present (2).

**110.** **Ectopterygoid/pterygoid boss, transverse width:** approximately as wide mediolaterally as long anteroposteriorly (0); >1.5 times as wide mediolaterally as long anteroposteriorly (1).

**111.** **Shape of the mandible seen in dorsal/ventral view:** bowed medially anterior to glenoid (0); not significantly bowed (1).

**112.** **Mandible, symphysis length as measured by the number of alveoli adjacent to the symphysis [relative to the number of maxillary teeth or an estimate thereof]:** long symphysis, number of alveoli adjacent to symphysis equals *c*.0.4–0.5 of maxillary alveolar count (0); intermediate, ~0.20–0.30 (1); very short, only 1–2 alveoli adjacent to symphysis (2).

**113.** **Shape of the mandibular symphysis in ventral view:** tapers anteriorly (0); laterally expanded (1).

**114.** **Structure of the dentary along the ventral surface of the mandibular symphysis:** no ventral elaboration (0); forms raised ventral platform or sharp keel/ridge adjacent to symphysis (1).

**115.** **Contributions to the coronoid eminence laterally:** surangular mainly (0); dentary mainly (1).

**116.** **Length of retroarticular process:** shorter than or subequal to glenoid anteroposterior length (0); longer than glenoid (1).

**117.** **Orientation of glenoid:** articular surface faces dorsally or only slightly dorsomedially (0); strongly inclined dorsomedially (1).

**118.** **Mandible, posterior opening of Meckelian canal on medial surface [anterior margin of the adductor fossa]:** dorsoventrally low with V-shaped outline in medial view [anterior margin of adductor fossa 'poorly defined'] (0); dorsoventrally tall [anterior margin of adductor fossa 'well-defined'], occupying at least half the height of the mandible with curving outline in medial view (1).

**119.** **Mandible, prearticular/splenial-angular contact perforated by anteroposteriorly elongate, oval foramen [lingual mandibular fenestra]:** present, emarginates the medial wall of the angular dorsally (0); absent (1).

**120.** **Rounded medial flange formed by articular and prearticular anterior to the glenoid fossa in dorsal view:** present (0); absent (1); absent but anterior part of outline of glenoid in ventral view appears ‘squared-off’ (2).

**121.** **Mandible, prominent longitudinal trough occupies much of the lateral surface anterior to the glenoid [dentary, angular, surangular]:** absent (0); present, bounded ventrally by robust longitudinal, ventrolateral ridge (1).

**122.** **Mandible, retroarticular process, dorsoventral orientation of long axis:** posterodorsal (0); posteroventral or subhorizontal (1).

**123.** **Mandible, retroarticular process, mediolateral orientation of long axis:** directly posterior, in line with ‘anteroposterior’ long axis of glenoid (0); inflected slightly posteromedially (1).

**124.** **Mandible, dorsal rim of 'lingual mandibular fenestra' formed by:** prearticular (0); splenial (1); at prearticular-splenial contact (2); inapplicable, lingual mandibular fenestra absent (?).

**125.** **Splenial participation in mandibular symphysis:** does not participate (0); participates (1).

**126.** **Angular relative length and participation in mandibular symphysis:** short, extends less than half mandibular length (0); long, extending more than half mandibular length, but does not participate in the symphysis (1); very long, participates in symphysis (2).

**127.** **Surangular, fossa and longitudinal crest on medial surface anterior to glenoid:** prominent longitudinal crest forms ventral margin of deep, dorsomedially facing surangular fossa (0); prominent longitudinal crest forms medial margin of mediolaterally expanded dorsal surface of surangular bearing shallow, dorsally facing fossa (1); crest and surangular fossa weak or absent, dorsal portion of surangular 'blade-like' (2); dorsolaterally facing fossa bounded laterally by a sharp crest (3).

**128.** **Coronoid length and morphology:** long, approaching or participating in symphysis (0); small, superficial element that is often disarticulated and thus not preserved, but represented by a facet on the surangular (1).

**129.** **Prearticular, large dorsomedian trough or rugosity:** absent or weak (0); present (1).

**130.** **Articular, deep anteroposteriorly oriented cleft [notch] posterior to glenoid:** absent (0); present (1); cleft absent, but dorsal surface is strongly concave mediolaterally (2).

**131.** **Number of premaxillary teeth:** four (0); five (1); six (2); seven or more (3).

**132.** **Regularity of posterior premaxillary dentition:** homodont, distalmost alveolus similar size to more mesial alveoli (0); heterodont, reduced distalmost alveolus (1).

**133.** **Regularity of maxillary dentition:** homodont (0); heterodont (1).

**134.** **Diastema at premaxillary-maxillary suture:** absent (0); present (1).

**135.** **Spacing between mesial alveoli:** narrow, less than mesiodistal length of a single alveolus (0); wide, more than half, or greater than mesiodistal length of a single alveolus and compact bone divides premaxillary and a small number of mesial dentary alveoli (1); one-third of dentary and more than one half of upper tooth row (2).

**136.** **Enamel ‘striations’ (grooves):** present (0); absent (1).

**137.** **Form of apicobasally extending enamel ridges:** coarse (0); fine (1); absent (2).

**138.** **Number of maxillary teeth:** 12–19 (0); 20–27 (1); ≥28 (2).

**139.** **Cross-sectional shape of teeth in anterior half of tooth row:** round or sub-rounded (0); sub-triangular [= trihedral] (1); intermediate between states 0 and 1, with a flattened labial surface, but this surface [is] not substantially expanded anteroposteriorly [ =  subtrihedral] (2); suboval (3).

**140.** **Premaxilla, diameter of first alveolus:** not significantly smaller than third alveolus (0); less than half the diameter of third alveolus (1).

**141.** **Relative neck length:** the neck is shorter [<0.8 times] (0); subequal to (1); or longer than [>1.2 times] trunk length (2).

**142.** **Axial rib articulation:** articulates solely with the axis centrum (0); articulates partly with the atlas centrum (1).

**143.** **Axial rib facet morphology:** double-headed (0); single-headed (1).

**144.** **Atlas-axis complex, atlantal centrum [odontoid], participation in anterior rim of atlantal cup:** does not participate, excluded by atlantal neural arch–atlantal intercentrum contact (0); participates (1).

**145.** **Atlas-axis complex, hypophyseal ridge:** absent or low bulge (0); present and prominent (1).

**146.** **Atlas-axis complex, hypophyseal ridge morphology:** longitudinally elongate ventral ridge of approximately equal prominence for its entire length (0); substantially more prominent anteriorly, forming an anteroventral eminence (1); located posteriorly (2).

**147.** **Atlas-axis complex, hypophyseal ridge location:** extends across both atlantal centrum, and axial centrum (0); does not contact the axial centrum (1); inapplicable, hypophyseal ridge absent (?).

**148.** **Atlas rib/rib facet or rib-like projection:** absent (0); rib present, contacts atlas *via* a distinct rib facet [sometimes co-ossified to atlas] (1); posteroventral projection resembling a fused atlantal rib, but lacking evidence of a rib facet (2).

**149.** **Axial intercentrum, size:** small, restricted to ventral surface of atlas-axis complex (0); large, wedge-shaped element that extends dorsally (1).

**150.** **Axial neural spine:** transversely narrow (0); transversely broad (1).

**151. Axial-atlas-axis complex, length:height ratio of centra:** <1.1 (0); 1.15-1.45 (1); >1.5 (2).

**152.** **Number of cervical vertebrae:** <15 (0); 18–23 (1); 24–29 (2); 30–36 (3); 37–49 (4); 50–59 (5); >60 (6).

**153.** **Proportions of anterior–middle cervical centra:** substantially shorter than high [length <0.7 x height] (0); approximately as long as high (1); substantially longer than high (2); even longer, corresponding to the ‘can’ shaped morphology of Otero et al. (2016a) or ‘elongate’ morphology of O’Keefe and Hiller (2006).

**154.** **Lateral surfaces of anterior cervical centra:** longitudinal ridge absent (0); present (1).

**155.** **Cervical centra, ventral notch:** absent, centra subcylindrical (0); present, centra 'dumbell’ or ‘binocular’ shaped (1).

**156.** **Cervical vertebrae, subcentral foramina and foramina on the dorsal surface of the centrum, within the neural canal:** both absent (0); both present (1); dorsal foramina present, but subcentral foramina very small or absent (2).

**157.** **Anterior cervical neural spines, morphology:** curve posterodorsally (0); inclined straight posterodorsally (1); inflected anterodorsally (2); inapplicable in some pistosaurians that have extremely low neural spines (?).

**158.** **Posterior cervical neural spines, morphology:** curve posterodorsally (0); inclined straight posterodorsally (1); inflected anterodorsally (2); inapplicable in some pistosaurians that have extremely low neural spines (?).

**159.** **Posterior cervical neural spines, height relative to centrum:** substantially shorter than centrum (0); subequal (1); substantially taller [equal to or greater than 1.2 times centrum height] (2).

**160.** **Rib facets of the anterior–middle cervical vertebrae:** rib facets broadly separated (0); two co-joined rib facets (1); mixture of single- and double-headed anterior cervical ribs (2); one rib facet (3).

**161.** **Rib facets of the posterior cervical vertebrae:** rib facets broadly separated (0); two co-joined rib facets (1); one rib facet (2).

**162.** **Cervical rib facets location:** ventrolaterally on centrum, do not contact neural arch peduncles (0); more dorsally, contact neural arch peduncles (1).

**163.** **Cervical ribs, size and orientation of distal processes:** marked anterior and posterior processes throughout cervical rib series, combined long axis of processes oriented approximately anteroposteriorly (0); processes reduced, especially anterior process, combined long axis oriented posteroventrally (1); large, anteroposteriorly expansive, sheet-like ribs with prominent processes (2).

**164.** **Cervical zygapophyses, combined width:** broader than the centrum (0); subequal to the centrum (1); or distinctly narrower than the centrum (2).

**165.** **Cervical centra, median ventral surface:** approximately flat or convex (0); bears a rounded midline ridge [*cf*. Tarlo, 1960] (1); or bears a sharp keel (2).

**166.** **Cervical centra, paired lateral ridges on ventral surface:** absent (0); present (1).

**167.** **Cervical zygapophyses, orientation:** horizontal (0); dorsomedially facing (1).

**168.** **Cervical zygapophyses, median contact between left and right zygapophyseal facets:** absent for most/all of length (0); present for most of anteroposterior length (1).

**169.** **Cervical zygapophyseal facets:** planar (0); transversely concave/convex (1).

**170.** **Dorsal and posterior cervical neural spines, dorsoventrally elongate groove on posterior surface:** absent (0); present (1).

**171.** **Cervical vertebrae, proportions of anterior cervical neural spines:** taller than their anteroposterior length (0); longer than tall (1); anteroposteriorly short and 'rod-like', approximately as long anteroposteriorly as the transverse width (2); as long as tall (3).

**172.** **Cervical vertebrae, shape of neurocentral suture in anterior–middle cervical vertebrae in lateral view:** rounded, ventrally convex (0); V-shaped (1); extends far ventrally so that neural arch contacts dorsal part of rib facet (2); extends far ventrally but is evenly convex and does not contact rib facet (3).

**173.** **Cervical centrum, proportional width:** mediolateral width subequal to height or less (0); at least 1.2 times as wide mediolaterally as high dorsoventrally (1).

**174.** **Cervical neural spines, apices of posteriormost cervical and anterior dorsal neural spines:** weakly expanded and convex (0); apex transversely expanded into prominent spine table (1).

**175.** **Anterior cervical centra, small, semi-oval ‘lip’ extends ventrally from anterior articular surface:** no (0); small, transversely narrow lip (1); broad, prominent lip, at least 0.5 times the width of the centrum (2).

**176.** **Posterior cervical rib facets:** face laterally (0); or posterolaterally (1).

**177.** **Middle­ to posterior dorsal transverse processes, distal articular facet:** dorsoventrally tall oval, perhaps composed of two weakly divided rib facets (0); composed of only a single subcircular facet (1).

**178.** **Height of dorsal neural spines in lateral view:** less than or equal to the height of the centrum (0); conspicuously taller than the centrum (1); more than twice as tall as the centrum (2).

**179.** **Number of dorsal vertebrae:** 17–19 (0); 20–23 (1); >24 (2).

**180.** **Number of pectoral vertebrae:** 2–4 (0); 5–7 (1).

**181.** **Dorsal neural arch height:** tall, base of transverse process located dorsal to midheight of neural canal (0); short, transverse process adjacent to neural canal (1).

**182.** **Dorsal transverse processes, orientation in middle dorsal region:** approximately horizontal [laterally oriented] (0); inclined significantly dorsolaterally (1).

**183.** **Dorsal neural spines, strong anteroposterior constriction at base:** absent (0); present (1).

**184.** **Dorsal neural spines, mediolateral width of apices in mid–posterior dorsal neural spines:** unexpanded, transversely narrow relative to anteroposterior width (0); mediolaterally thick, subequal to anteroposterior width (1); alternate spines expanded laterally to one side (2).

**185.** **Posteriormost dorsal rib facets:** prominent transverse process located entirely on neural arch (0); rib facet split between neural arch and centrum [‘sacralised’], but bears a typical posterior dorsal rib (1).

**186.** **Sacral ribs:** cylindrical and slightly expanded towards distal end (0); transversely expanded, dorsoventrally thin and sheet-like (1).

**187.** **Caudal vertebral count:** 25–30 (0); 33–35 (1); 36–40 (2); >40 (3).

**188.** **Caudal ribs facet location in proximal–middle caudal vertebrae:** located dorsally, contacting or almost contacting neural arch (0); placed dorsally but neural arch does not form part of facet (1); at midheight of centrum or lower (2).

**189.** **Caudal centra, outline of middle caudal centra in anterior view:** suboval (0); subrectangular, chevron facets widely spaced and located ventrolaterally, ventral surface approximately flat giving a subrectangular appearance to centrum in anterior view (1).

**190.** **Caudal centra, length:height ratio of proximal caudal centra:** >0.85 (0); 0.6-0.8 (1); <0.55 (2).

**191.** **Caudal centrum, subcentral foramina on ventral surface:** paired lateral foramina (0); single midline foramen (1).

**192.** **Caudal vertebrae, chevron facet:** located equally on anterior and posterior edges of the centrum (0) or mainly on the posterior edge, low, mound-like eminence may be present on ventrolateral surface of centrum anteriorly (1).

**193.** **Middle and distal caudal vertebrae, chevron facets:** flush with level of ventral surface of centrum (0); project significantly ventrally (1).

**194.** **Proximal caudal centra: width to height ratio:** 0.9-1.1 (0); >1.1 (1).

**195.** **Distalmost caudal vertebrae, forming ‘pygostyle’:** absent (0); present (1).

**196.** **Ratio of coracoid to scapular length:** > or equal to 1.9 (0); 1.6–1.9 (1) < or equal to 1.6 (2).

**197.** **Anteromedial margin of the coracoid:** does not contact the scapula (0); contacts the scapula (1).

**198.** **Anteromedial margins of the coracoids:** do not contact the dermal girdle elements [clavicle and interclavicle] (0); contact the dermal girdle elements (1).

**199.** **Scapula morphology:** dorsal blade expanding ventrally to form acetabular region, lacks expanded ventral plate (0); triradiate with expansive ventral plate (1).

**200.** **Contact of the ventral plates of the scapulae along the midline:** do not meet along the midline (0); meet along the ventral midline (1).

**201.** **Scapula blade, outline of anterior margin in lateral view:** approximately straight, weakly concave or weakly convex (0); pronounced posterodorsal inflection (1); distinct concave region anterodorsally (2).

**202.** **Shape of the anterolateral margin of the scapula where the dorsal ramus meets the ventral ramus:** flat or gently convex (0); forms prominent ridge or shelf (1); inapplicable, ventral plate not developed (?).

**203.** **Scapular blade, anteroposterior width at distal end:** subequal to width at midlength (0); narrow, tapering dorsally (1); broad, distal part expanded relative to midlength (2).

**204.** **Scapula blade, length relative to posterior process of scapula:** blade longer (0); blade subequal to or shorter than (1).

**205.** **Scapula blade, angle relative to ventral scapula margin:** vertical/subvertical [70-90 degrees] (0); intermediate posterodorsal inclination [45-60 degrees] (1); strong posterodorsal inclination [30-45 degrees] (2).

**206.** **Scapula blade, medial surface:** smoothly convex or flat (0); robust buttress oriented parallel to long axis of blade (1).

**207.** **Coracoid, posterolateral cornu:** does not extend as far laterally as glenoid (0); extends to level of glenoid (1); extends lateral to glenoid (2).

**208.** **Coracoid, median fenestra/large embayment:** absent, although intercoracoid contact may be slightly split posteriorly (0); median embayment present (1); posterior processes strongly divergent forming prominent V-shaped or otherwise mediolaterally narrow emargination (2).

**209.** **Coracoid, shape of anterior process:** anteroposteriorly long and transversely broad, approximately rectangular (0); anteroposteriorly long and transversely narrow (1); anteroposteriorly short and subtriangular (2).

**210.** **Coracoid, posterior margin, outline in dorsal view:** oriented approximately mediolaterally, may be convex, straight or weakly concave (0); anterolaterally oriented (1); possesses a distinct posterior process adjacent to midline (2); oriented posterolaterally (3).

**211.** **Coracoid plate, perforations/large foramina:** absent (0); present (1).

**212.** **Coracoid, dorsoventral height of anterior process:** dorsoventrally low and thus plate-like (0); taller dorsoventrally than mediolaterally (1).

**213.** **Coracoid, anterior process orientation:** extends approximately anteriorly (0); inflected anterolaterally (1).

**214.** **Coracoid, robust buttress on dorsal [visceral] surface connecting glenoid to median symphysis, orientation:** posteromedially (0); mediolaterally, and forms posterior margin of an anterior depression (1); mediolaterally oriented, but located anteriorly so anterior depression is absent (2).

**215.** **Coracoid, ventral projection/process extends from intercoracoid symphysis:** absent (0); present (1).

**216.** **Coracoid, low, mediolaterally oriented buttress connecting glenoid to median symphysis on ventral surface:** present (0); absent (1); mediolaterally oriented shelf or sharp crest extends anteriorly from coracoid surface bounding pectoral fenestra posteriorly (2).

**217.** **Clavicle/interclavicle complex, median fenestra:** absent (0): present (1).

**218.** **Contact of the clavicles along the midline:** present (0); absent (1).

**219.** **Clavicle/interclavicle complex, shape of anterior margin:** markedly concave, mediolateral width of concavity at least 1.25 times the anteroposterior depth (0); anteriorly convex or pointed (1); transversely broad and almost straight (2); small, transversely narrow, weakly concave region (3).

**220.** **Median pelvic bar:** present (0); absent (1).

**221.** **Ilium curvature shaft in lateral view:** appears straight, pelvic articular end equally expanded anteriorly and posteriorly (0); curves anterodorsally, articular end expanded only anteriorly (1); sigmoidal (2).

**222.** **Ilium, rotation of dorsal blade relative to long axis of proximal articular end:** ends perpendicular to one another (0); ends rotated by approximately 45 degrees relative to one another (1).

**223.** **Ilium, shape of dorsal expansion:** subequal anterior and posterior expansion, occupies dorsal half of ilium (0); subequal anterior and posterior expansion (or narrowing) confined to dorsal one-third of ilium (1); asymmetrical, extends much further posterodorsally than anteriorly, dorsal surface slopes posterodorsally (2); inapplicable, dorsal expansion absent (?).

**224.** **Ilium, anteroposterior width of dorsal expansion:** tapering, less than minimum shaft width (0); slight, just greater than minimum shaft width (1); expanded, between 1.5–2.0 times the minimum anteroposterior width of the shaft (2); large, over 2.5 times minimum shaft width (3).

**225.** **Ilium, tubercle on posterior surface around midlength:** absent (0); present as a tubercle (1); rugose, proximodistally oriented crest present (2).

**226.** **Ilium, cross section of shaft around midlength:** subcircular (0); mediolaterally compressed, suboval (1).

**227.** **Ilium ratio of length to minimum anteroposterior width:** <3.0 (0); 4.0–5.2 (1); >5.3 (2).

**228.** **Ilium, pronounced, broad fossa on medial surface of the dorsal end:** present (0); absent or weak (1).

**229.** **Pubis, ratio of anteroposterior length to minimum mediolateral width:** < or equal to 1.2 (0); >1.3 (1).

**230.** **Pubis, anterolateral cornu:** absent or weak and rounded, extending less far laterally than acetabulum (0); or present, extending further laterally than acetabulum (1).

**231.** **Ischium, length to width ratio:** < or equal to 0.9 (0); 1.0–1.3 (1); 1.4–1.8 (2); >1.85 (3).

**232.** **Limbs, postaxial accessory ossicles:** absent, or small, round elements appearing late in ontogeny without well-defined articular surfaces (0); present as small elements (1); present as large, well-defined elements contacting other limb bones (e.g. humerus, ulna) *via* well-defined articular surfaces, ossification of these elements is often late but their presence can be inferred by the presence of articular surfaces (2).

**233.** **Second postaxial accessory ossicle articulating with propodial:** absent (0); present (1).

**234.** **Limbs, preaxial accessory ossicles:** absent (0); present but small (1); present as large, well-defined elements contacting other limb bones (e.g. humerus, radius) *via* well-defined articular surfaces, ossification of these elements is often late but their presence can be inferred by the presence of articular surfaces (2).

**235.** **Forefin, ratio of proximodistal length excluding humerus to maximum anteroposterior width of humerus/proximal epipodials [aspect ratio]:** <3.0 (0); 3.1–3.5 (1); >3.6 (2).

**236.** **Ratio of forelimb length to trunk length:** <0.8 (0); >0.9 (1).

**237.** **Difference in the axes of propodial and rest of the paddle in the forelimb:** proximodistal axis of digits and tarsals/carpals collinear with propodial long axis (0); digits/tarsal/carpal axis extends posterodistally relative to propodial long axis because proximodistal length of radius/tibia is substantially greater than that of the ulna/fibula (1).

**238.** **Propodials, dorsal and ventral surfaces of distal half:** uniformly convex or flat with robust pre-and postaxial margins (0); weakly concave region separates central, convex portion from strongly tapering, flange-like pre- and postaxial margins (1).

**239.** **Humerus, long axis curvature in anterior view:** straight or almost straight (0);

**240.** **Femur, long axis curvature in anterior view:** straight or almost straight (0); pronounced dorsodistal curve (1); pronounced ventrodistal curve (2).

**241.** **Ratio of humerus to femur length:** <0.85 (0); 0.9–1.1(1); >1.1 (2).

**242.** **Epipodials, ratio of radius to tibia length:** <0.89 (0); 0.9–1.09(1); 1.1–1.3 (2); >1.4 (3).

**243.** **"Tongue-and-groove" articulation between propodial and epipodial:** absent, distal articular surfaces of propodials smooth (0); present, deep recesses in distal articular surfaces of propodials accommodate highly convex proximal surfaces of epipodials (1); absent, but a prominent anteroposteriorly oriented ridge bisects the epipodial facets (2).

**244.** **Humerus length *versus* width ratio:** >2.9 (0); 2.3–2.7 (1); 1.7–2.2 (2); < 1.6 (3).

**245.** **Preaxial margin of distal humerus in dorsal or ventral view:** straight or convex (0); concave [distal humerus expands anteriorly], but anterior expansion relatively small, substantially less than posterior expansion (1); concave, and anterior expansion is large, approaching the size of the posterior expansion (2).

**246.** **Sharp longitudinal ridge on anterior margin of humerus:** absent (0); present (1).

**247.** **Shape of the distal end of propodials:** uniformly convex (0); propodials distinctly angled for articulation with the epipodials (1).

**248.** **Humerus, angle between long axes of epipodial facets in dorsal view:** oblique (0); close to 180 degrees (1).

**249.** **Humerus, inclination of proximal end in dorsal view:** inclined posteriorly so that the proximal portion of the anterior margin is convex in dorsal view [often a low mound is located proximally on anterior surface] (0); not inclined, extends proximally so shaft appears straight (1); inclined anteriorly so shaft appears sigmoidal (2).

**250.** **Humerus, shallow groove on ventral surface between epipodial facets (flexor groove):** present and prominent (0); present but anteroposteriorly short and shallow (1); absent (2).

**251.** **Femoral length *versus* width ratio:** >2.8 (0); 2.1–2.7 (1); 1.55–2.0 (2); < 1.5 (3).

**252.** **Humerus, tuberosity morphology:** narrow and projects dorsally (0); broad and projects posterodorsally [tilted] (1).

**253.** **Femur, trochanter morphology:** narrow and projects dorsally (0); broad and projects slightly posterodorsally [slightly tilted] (1).

**254.** **Ratio of radius length to maximum width:** >2.7 (0); 1.1–1.5 (1); 0.8–1.0 (2); < or equal to 0.75 (3).

**255.** **Ratio of tibia length to maximum width:** >2.5 (0); 1.1–1.8 (1); 0.8–1.0 (2); < or equal to 0.75 (3).

**256.** **Radius morphology:** preaxial margin concave (0); straight or convex (1).

**257.** **Radius, prominent anterior flange extends from anteroproximal surface:** absent (0); present (1).

**258.** **Ulna morphology:** postaxial margin concave (0); convex (1).

**259.** **Tibia morphology:** preaxial side of tibia concave (0); convex or straight (1).

**260.** **Ulna, expansion of distal end relative to shaft:** absent or very weak (0); present (1).

**261.** **Epipodial foramen [=spatium interosseum; =antebrachial foramen]:** present, proximodistal length slightly shorter than epipodials (0); present but short, proximodistal length less than half epipodial length (1); absent (2).

**262.** **Ratio of maximum radius length to maximum ulna length:** 1.0–1.3 (0); 1.4–1.7 (1); > or equal to 2.0 (2).

**263.** **Radius, posterodistal facet for intermedium:** absent (0); present (1).

**264.** **Tibia, posterodistal facet for intermedium:** absent (0); present (1).

**265.** **Width of epipodials of the hindlimb:** tibia larger (0); widths within 10% of each other (1); fibula larger (2).

**266.** **Anterior (I) and central (II) distal tarsals/carpals:** offset relative to proximal tarsals/carpals so distal elements articulate with multiple proximal elements (0); in line with proximal tarsals/carpals, lacking anteroproximal or posteroproximal articular surfaces (1).

**267.** **Interlocking distal phalanges:** absent, digits splayed (0); present, digits parallel (1).

**268.** **Position of the fifth metapodial:** lies in the metapodial row (0); shifted proximally so that the proximal half is in the distal mesopodial row (1); shifted proximally so the entire fifth metapodial is in the mesopodial row in manus (2).

**269.** **Metapodials, morphology of proximal ends:** all metapodials form straight, anteroposteriorly oriented butt contacts with distal tarsals (0); at least one metapodial possesses a bifaceted proximal articular surface (1).

**270.** **Phalanx proportions:** long and slender [~2-3 times as long proximodistally as broad anteroposteriorly] (0); short and robust (1).

(b) Matrix in TNT format

xread

270 129

Neusticosaurus_pusillus 00000010000000000???0000000020000?000000?000010??00000001?00?00?00?????????000????????00?0000000???1?0??0???0?0200100?00010?10?0003000?12000010?0??000011000??000010000000100000000?1000?03000?0000200000110200?00000?0100?????00011001000?000001200100??20??000000000000?00?0

Nothosaurus_marchicus 11000021110?00?00??020002100200000001??110000?0??000001?1?0??10?000??1001??001????????1000100000???1?0??0???101110?10?00010?00???010100121010??????????11000??0??110000000100001000?1000?0?????????[0 1]00000110100?00000?0100?????00011001000??00001100000??20??000000000000?0???

Augustasaurus_hagdorni 010100?0010?00000??00100100000000000000100?0010012101000010??????0???0?0000??????02??000?0110000?0010000????0?1100?10??1001??1???01010002100???00??21??41?01??000000200?0?10000011??0000???????????00?000?102?020000?0?????????????????000??00???100000000???0?100?1000???0?00

Bobosaurus_forojuliensis ????????????????0??????????????????????????????????????????????????????????????????????????????????????????????????????????????????????02?0?00?00??01?2100000??110?12?000?00??001221100?10?1????????????????????????????????0122010?001??????0???1?000????????0???0?0??0??????

Pistosaurus_longaevus 010100?0010000000??001001000000?10000001000001001200?000????0010?0???????????????02??00000??0000?0010020????20????????????????????[1 2]0100??00?????????????20011100000000000110100101100010????101110?00?001?202100??0??001????0122210???1??????01?1?00000000???0???0?10????????0

Yunguisaurus_liae 0001000??10000000??0010010?000010010?000?000110??200??00?????01020???????????????02?10???????0?0?0010020???10011001100?1001?1????13??0??????1??????????41000??00?000000?001??0??01210?0?00[2 3]??0??????0?000?200????????????0?00122011?0000002000??11000?000?0?000000010000100000

Abyssosaurus_nataliae ????????????????????????????????????????????????????????????????????????????????????????????????????????????????????????????????????????????2???1110??1?1001???3201200111??01?01??1011??0???0???????0010000100??2??10?0?001?1?1101110??2?1??1????10???10?22?0?3??110???1001211

Acostasaurus_pavachoquensis 11?1?0?0211?10210??010110?00210?0100???????00?0103112????201?00?1000012?1?10100???2?1??0?1101010?0210?3?1?????111011110001111120?00011010000?1110??0??0?0001???1?01110000??00?0???????????????????????????????????????????????????????????????????????????????????????????????

Albertonectes_vanderveldei ????????????????????????????????????????????????????????????????????????????????????????????????????????????????????????????????????????????2?1?1101?026311122232022001111101001110?1100?01200?11112001110110?[1 2]1??????10???11?11?01001?0002000??12021010122?12210110[0 1]0110[0 1]1211

Anguanax_zignoi ????01?0?????????????????????????1???????????????????????????0?????????????????????????????????????????????????????11????1?????????????1??0????0????1???0?01?????0??10?????????????????????00??????????????????????????????????????????????????0??0???00??1????1?1?0?0????1??0

Anningasaura_lymense 111000?0120?00??10000100?0010000100000?10000010112001000?10?0?1020?0110111010010?00211?1000000011?00?000?111??1110?100?1010?110??00010011000?0?00??0?0??10010??3?0?120100?031?1???????????????????????????????????????????????????????????????????????????????????????????????

Archaeonectrus_rostratus 1002002???0?102?1111??00?????????0?000?10000010012011000?1??01?????????????????????????????????????????????????11??1????1?????????????11120?0??????????2100100111000201???0?11?0110110110010??????0?????0?20?1??????????????01?2?1100?10001000??1?020?00??11011011010001101100

Aristonectes_parvidens 00?010??1?0000201??00200200000??00?????11??01010?3212?1?0000?00?1?01212010???????????????0?10000??????????????0101010??1000???????3000011200?1101101?02?1111???3???200111??01?0???????????????????????????????????????????????????????????????????????????????????????????????

Aristonectes_quiriquinensis 00??1?0?????0????????????????????????????????????3???????????00?1????120?0????0100?????????????????2?110???????201?10???000???2??0??0?01123?1?10???1?014111122132022001111?0100????????????201??01?20?1100111011200?021????????????????100?1000???03201112?1?1?101?00011?01210

Arminisaurus_schuberti ??????????????????????????????????????????????????????????????????????????????????????????????????????????????0???010??1011???2?01?????10?0?????????????1001?1?11011[1 2]01001?001[0 2]010??0100?0?0110001????1?010010???????????????????????????????0????0???00????????????0????????0

Attenborosaurus_conybeari 00010010???110??112???0010?0?????0??0001002??????2??10000???11??????????????????1?2???????????????????????????0110??0???0?????????10100???0?2??????????310??011110????1???0?00??11101100?0???1???1?20?10?1????0220000?000?310?02??1?0010001000??1??10?00?????1?001?100???01101

Atychodracon_megacephalus 100101212201121010002100100100?000?000010000010012001000110101???0?201?000???0??1002111000100011011???00?0002001111?0??20?0?11??0??00001020?0???0???10120001???1100?201????10?00111?1012001??20?0?0??????????1??????????????01022110????????00??1?0201001?110?1??1010??0?????0

Avalonnectes_arturi ??????????01??10????????10?????01??00001000001001100100001010???????????????????1??????0???????????????????????????????????????????????????????????????2100?00211001??100?0110001101101200?0110?00????1?01201???????????????0102?110??1?????????11020?000?110110?101000010?100

Borealonectes_russelli 11010?1?210?102011100?00?0?0000??010?0010010000????1?000??0?0???20???12000???????022?1?0???????0??2???201?????111??00???1?0?110??0??1?01?10?00100??21?120001???2?0??20?????30?0????????1???????????????????????????????????????????????100??00?????1101012???2?011?0001???11?1

Brancasaurus_brancai_composite_SHK 00???0?0?0?????0102???0011??????0??????1?010??02??3?1?1?110?001101?[0 1]1???1???????0022???????1???????????????????????100?1110???2??1??????????2010???10?[1 2]410010[0 1]23201210101110[0 1]0[0 2]1110011[0 1]0000[1 2]011101[0 1]???1??1??20???????210?0?010121021010??????0111?02001022210???????0????????0

Brachauchenius_lucasi 00020120211100210??010001000210?111?0001012001000312100012??00???????????????????011?0100?1011?0??2100310???211???001?00001?11?????????10?0?0???0?????000002????211?00?????????00??001???????????????????????????????????????????????????????????????????????????????????11111

Brachauchenius_MNA_V9433 0102?????1?100210??0100010?0????????0001???00?002?1210?0?20?0????0?????????????????????00????110??2110210???2111000010?0001?1110000000110000???????????0000211?3211100000?200?2?0???01??????????????0?1???????22200002?????11??0011?10????????????????????????????????????????

Callawayasaurus_colombiensis 000010?0?10000??10100?0020?000?10????????01010000220101010001111011??02?1010??1?002211001????0?0???2?1100???1?1101??0??20?????2??010000110012??????????5210102232022001111?0100111211110???????????200100?11001120010?10?01?????????00??0???100?220300102?3101200?10?0111??2?1

Colymbosaurus_trochanterius ???????????????????????????????????????????????????????????????????????????????????????????????????????????????????100?1000??????0????????????1?1110??2410012??320?200111??01?01??1011??00?2010101??0???????????2??102?2???????????????201??10111?2210101221022101102?11101211

Cryonectes_neustriacus 10??????????1[0 1]??0??0??00?????????????????????????????????????0????????????????????2011???????1?10121?00??000??1?01?10010010?1110?011?01???01????????????1001???1?0??00???0?0[0 1]?0???????????????????????????????????????????????????????????????????????????????????????????????

Cryptoclidus_eurymerus 00100?0010?101100??00100101000010000020100201001011010000000010?1011101101?111011111??00101100011?00?0????000?02001100?1000?0021?0200001110121111101?013100111232011001110000000111011000000010001121011001010201201020200?1100210110112010?101113032010122101300110?2??001211

Djupedalia_engeri ??????0?????0?????????????????????????????????????????????00???????0?01111????????????????????????????????????020??????????????????00?0??1??2011???100?510012113201200111030100110??1100??????????????11?0???0?0??01??????3????????????2?0??????1?02?0?012?1???101102???1?1211

Dolichorhynchops_bonneri 00120120100000210??010000110001?001001010021???223010111010?110??1?0?1?????1????0013111?10?100011122?020010[0 1]0?10000010011?101220101000111200?110120010110001111320?120100?000?10?1101100??001??100?101101?002020111?02??101100110?110?12100101??111300102231033101101011001[1 2]11

Dolichorhynchops_herschelensis 00110?200000002011101000[0 1]?10001?001????????1???223010111?100110?01??1?10100???????1311???0??0??11122?02001?10?10000110?10010120?1110002112010??????????100010?23201120100000010010[1 2]?110?00?011?100?21110??????201110020110111?110?21113???????????????????????????????????????

Dolichorhynchops_osborni 0012012011000020111010000110001?001001010021???22311[0 1]1110100110?01102?201001?01100131111100100011122?01001010?10000110011010120011100021120?011010001021000101232011[1 2]010000001001120110?0000???11??101101000002011100201101110110121113210100000101200102211033101101011001[1 2]11

Dolichorhynchops_ROM_29010 00?[1 2]???0????00[1 2]?1110??00???0???????????????????223??01???10???0?0110211010010011001311??1?????0111?2?020?1?1???00???????????12?0??1000111?0101101000?02?00011123201120100000011011?00100???0120101????1?110020??????????1010????????11?210???10???13101022?1?3?101?0101?001??1

Dolichorhynchops_tropicensis 00120?20???0002?11101000?1?0001?0010???????1????2[2 3]110111?10??1???????????????????01311??1????0?11122?02001?00??000??????1???12?0??100?211[1 2]0????????????[1 2]1001???320??201?0????1?01?20?1?00?????000??1011001002020111012?????110111?211132?0??0???111200102?2103310110101100121?

DORK_G_1_2 ?????????????0?????????????????????????????00100231210???20?0?????????????????????????????????????????????????????????????????????????110?0???????????????????????????????????????????????????????????????????????????????????????????????????????????????????????????????????

Eardasaurus_powelli 1001012??11110210??0100010?0001??10?00010020010?0?1211?00???0010?0????????????????2211???????????12110300?????1111?110000001111000[1 2]1110102000??00??2??01000[1 2]112120011010000001100020110??1?0???00??????????????????????????????????????200??0?000002100012?0022101110011111111

Edgarosaurus_muddi 100101?021001020111010001??0?0??001000010?200102220011101000111101?02?2?1?0????1002211?01??????10122?13001002011000110?1111?120011[2 3]110111100?010100000121001?1132011201000000000???01????????????????????????????????????????????????????00?00??????0010?2?0?3?10??0101??01211

Elasmosaurus_platyurus ?????????????0??10[0 2]????0????????????????????????????????????????0??????????????????????????????????????????????100?????????????????0??011??1201?1001?0263111?22320?2101?????1???11??1110???20011?1??10110?1100??1??102?????01?120?1??01???????????????????????????????????????

Eoplesiosaurus_antiquior ????????????????????????????????????????????????????????????????????????????????????????????????????????????????????????????????????????????2??????????4100?0111?0?12?1???03??0??????????01000?11?02??1001002?0?0??00???????0??2?11?00110020000?11010100??1?0110?1010000101100

Eopolycotylus_rankini 00??????????10????????00???[0 1]???????????????????????????????????????????????????????????????????????????????????000??????????11?0??1000110?0?????????????0?01?01?2?????1??1??00?01?2?110????????????0?1100?002020111?1???111110?00?21113210??01??1?12001022210?2???1????11011?1

Eretmosaurus_rugosus ????????????????????????????????????????????????????????????????????????????????????????????????????????????????????????????????????????????????????????100110?100?12010000?000111??11?0???0000?01????1?????????0??0???????0010201200011000000??11021?100020011011010000201100

Eromangasaurus_australis 000000???10000??10200?0020?000??00?000010??0010???????101?0?11????????????????????2????????????0???????????????1011?????010????????000011001?01?1101??2?2101???3?0??????????1?0???????????????????????????????????????????????????????????????????????????????????????????????

Eurycleidus_arcuatus ???????????????????????????????????????????????????????????????????????????????????????????????????????????????111??????????1?????????01110?????????????100100211000201000010100?1?1101????0110100?201101120010201000100?10001020110001??????0101?02110010110????1?10????????0

Futabasaurus_suzukii 00??000?1?0?00??0??00?0?2?0000????????????????????0???1??????????????????????????????????0110000???????????????100??????0?????????100001100???????????????11?22?2022001111?010?11110111000??????????????????????????????01301?1??01?0?1100200000220300102221122101100011101?10

Gallardosaurus_iturraldei ??0?01?022?1??21????????10??????11?0000100200100?3121100020?011021???1????????????121110???0?010?12110310???211?????1???0?????????????????2????00??2111?00011??1?0?110?0??20??????????????????????????????????????????????????????????????????????????????????????????????????

Georgiasaurus_penzensis 0012??2??0??00??11101000???0?0???0?????????????????????????????????????????????100??1????????0?11?????20?1?1??100?????????????????????[1 2]???0?0???????????0???????????????????????????????????????????????1?????????????????????????????????????????????????????????????????????

Hauffiosaurus_longirostris 00?20??0?1??12111120??00???2????????????????????02???????????01001??0???1?0?????1110?????????????110?0??0?????1001?100?1100?11?0023010011200??????????????????????????????????????????????????????????????????????????????????????????????????????????????????????????????????

Hauffiosaurus_tomistomimus 00020?10210?1?110??0100?0?0210100100???????00?00010100?0?101?01011??????1001????111011?0?0100010?100?010??????1000?100?1100?112002301001120?1100?????1231001?11110?100100002000011101010001??10???0???1??1?0?0????????????00????????00???0?1000012010000121?01101?1?0?11201?00

Hauffiosaurus_zanoni 00?2??1??1??????????????0?0??????????0?10???????????00?001???010????????????????11101101?0100010?100?010????20100??100?1100??12002301?01120000?00??????310011?11100100100?020?00?????????0?01100000201101120??0020000?010?0001020?2?0001001000??11010?00121??11011010001101100

Hydrotherosaurus_alexandrae 000010?0??0000201?100?002??0000?0011000100111?????2121?111??01?????????????????????????????????????????????????100010?1?01??112???10000110012???1?????1521112223202200111?101001110011100002011?11?1001?00001011200?020????120111011000100?000??12031010123102300110?011101211

Hydralmosaurus_serpentinus 0?0010?0??000???121?020020?0000?0??10??1001????0?3??2?111?0?11???10??12???????0???????????????????????????????????1?0???0?????????1???01?0??2??????????63111222320220011111?1001110?11100?02010111??0011???????12????2100?11101100?10?1100??000010?31010122103210110??110?1?10

Kaiwhekea_katiki 00001010??000???1020020020?000?????????11????0?0?32?2111000?00????????????????????????????????????????????????0101?10??1000??????0300001120?2??????????51011122320?2?0111?10100?11111?10???2????0??????????????????????????????????????000??00??11?32?11??3?12110100?0012?1??0

Kawanectes_lafquenianum ????????????????????????????????????????????????????????????????????????????????????????????????????????????????????????????????????????????????????????211122232022?01111?01001??00111000?2001001????1?001?1??????????????01???10??001?0????0??2?03101112310?3???101??11?1??1

Kimmerosaurus_langhami ??1??0?1??0?0????????????0?????10??0??????20100000102000000011101010201101?101111111?????????????200?0????????0200110001000?002??0?00?01220???1?1001?01?00011??3?01100111???1?0???????????????????????????????????????????????????????????????????????????????????????????????

Monquirasaurus_boyacensis ??????2???????2??????????0???????11?0??1???0010????2??????????????????????????????????????????????????????????????0?????????????????1??10?0?0??????????0?0?????3?1?????????0???0??10????????????????????????????????????????????????1??000??????00?110????1??2310????0??0????1

Kronosaurus_queenslandicus ??0?0?20?1??????0?????????????????????????????????????????01000?[0 1]?00011000?01110?111?0???????????12110[2 3]10?????1101000?10011?1120000?1?01010?011?0??2??00000?11?3211?0?000?0001??0?20?1??0??2?10000?00?10?0?????02??0020????1????????103??????????????????????????????????1????

Lagenanectes_richterae 01??????1???00??0??0???0??0???????????????????????????????????1101?????????????01?2211?0?0110000?12?????0???????01??????????11?0??1010011?01?0101101??1?11010??3?0?210111?0?0?0????????????201[0 1]101??????????????????????????10111021?????????????????????????????????????????0

Leptocleidus_capensis 010001?1112?1010102002001?0000?10??0000??01001022?201010?0??01??21?????0?????????0221110?01?00011122?12110001?????1110?0111???2?111111010101???????????210010??320?1?0100?0?0?0?????1??????????????????????????????????????????????????2????00????0???????????2???1?0??10?1???

Leptocleidus_superstes ??0?0??1?1?0????10??????1??????????????????0?10222201010?100011121?0011?01???????02211???????????122??31????1???????????????????????????????????????????0?01?0132011001000??00?110?11102???1011001?10110011020??1?100200102??????????????????01???02001012?0??????????????????

Libonectes_morgani_composite_SK 000000?011000[0 1]20112001001000000?0011??010011????0221??111?00010?010121201011000?1?22110010100000?122?1300???21110[0 1]01010200??01200010100110202010110111262111?22320121011110010011??0111?0??2120100?210?1???????110000?11001010?1?121?010001??100?10320101231?22100100?111?1210

Liopleurodon_ferox 11010120221110210??010001000101?11?0000100200000031211000?0100101100?1100001??101022111001101110?12110210???2111110010?0010?111000111101010001100??0110100011121200100101000011000?0110????2010100????1??0?000?????0??01???1????????10320???010???02101012?0?2?101?0001???1?11

Lorrainosaurus_keileni ??????????????????????????????????????????????????????????????????????????????????????????????????????????????0?11?01?10111?11??00????010?0????????????????????1??0???????????????????????????????????????????2220000101?????????????????????????????????????????????????????0

Luskhan_itilensis 0102012??21100210??0?00001?000???1?0000100200?0023120100010??010??10?11?10?1?11??01??????????????12110????????10000110?0001?113??03000?1??2001101??01?010001112321120000002011201???010????0010001?00?1??021002020000201????0023?1??1??000?001010102001012?0033000?02010211?11

Macroplata_tenuiceps 1101001??10?10101110??001??1001010?0???????0010????0?????????010100?012?100??01?100211???????????100??00??????1111??0??21???11??00201001110000100??210220001???110?120100??30?001?11100000?0110000?20110?1????0000000?001?0001?2012?002?????0?101?0?0?0?0?110?1???010??01?????

Makhaira_rossica 0??????????????????????????????????????????????????????????????????????????????????????????????????????????????100??????????11????20??010?10????????????????????????????????????????11???????????????????????????????????????????1????????????????????????????????????????????

Manemergus_anguirostris 00011020?1000020111010001??0001?001????????1???22[2 3]2?0111?10?11????????????????????????????????????22?0[2 3]00?????1000010???101?12????1000211?011??????????210010??3201?00????000???1?0011????????????????1????????0?10??????1??????????0?1??????0????????????[1 2]???????????????????

Maresaurus_coccai 110100?1220?1???1120?1001?0?00?????????????00001120?10???10?0110?0?????0?????????0??11?000101010?122?1000???200111?00??2100?11???01?1?010100????0???????0001???2?0????1???030?????????????????????????????????????????????????????????????????????????????????????????????????

Marmornectes_candrewi 0???????????00??0?????00???????????????????????0????10???0???0????????????????????????????????????????????????10100110?0000?111000201021020000000??21?1?0001???1200100100?200?1001??1?00?1?0010000??????1?0??000?00???00????00220111??2000??00110001000012100?1??1110??011???1

Mauisaurus_haasti ????????????????????????????????????????????????0[2 3]??21???1?00?1101?01?2????????1002????????????????????????????????10??1010???2??0?????????????????????62111?2232022001111?01001110?111000?20????1????1?001110?????????????11????0??0??000??00????031011123112110110[0 1]0111[0 1]1211

Mauriciosaurus_fernandezi 00????2??1?0????????????01????????????????????????????1??????1???????????????????01[2 3]11?110?0?0011122?1300110??1000??1???1???12??????0?2??[1 2]0?0??????????10?0111132011201???00??0???1????????????00??1??10????1?????1???0??????0?????????210?0?????0???????2????????????11??111?

Megacephalosaurus_eulerti 010200?0211100210??110000000210?111000010120010023121000120?00???????????????????011???00011000101211031010?2111000010?0001?1110000000110000???????????????????1??????????????????????????????????????????????????????????????????????????????????????????????????????????????

Meyerasaurus_victor 10?00?0?21??00??11??0100??0000???0??????????????????10?0??0101???0????????0??????022?0?1?01010111122?11000101?0111100?02110211???01?1001010?0???0???1?120?01?????00?20????0???0011211012002?110?0?0111?0?1????0201000?0001000102011?0011002100??12020?00021??11011010001101100

Microcleidus_brachypterygius 0011000?110102100??0010010000000101011110010010102001000100?????1???????????????101??100?0010000?12100000???20?????????????????????0000110012?????????2321011121?00??1????00100?12011110?03100011?0210???1????20130?0?01???101220?10??01002000??11021?100?2??11010010011101101

Microcleidus_homalospondylus 0000000011010210???0010020?000?010?0111100100101020010001??11110110010200001??001021?1?00????000??2100100???2001001??00?0???102???10000??0012??????????42101112100020110010010011201111010?1000011??1011110001?01?010101???00122012000010?20000011021?100110011011010011201101

Microcleidus_tournemirensis 0000000?120102100??0010010000000001011110010010002001000110?1????1?????000?????0112?110000??00?0?12100100???20????????????????????11000110012?????????142101112100?20?100?0010011200111010?????????201100120211013010?010?300122012000110????0??1?020?100?2?0110110100012?1??1

Muraenosaurus_leedsii 00????????0?01100??001001??000?10??????????010?0?????1???0???10?00?0????11?????11011??????????????0???????????0201010002000?0021?010100110012?1?1101??14210112232012001110001001??11111000?200?111???????0???0?????1?2020?3?1012?021??12????1001230311101221023001100111001210

Nakonanectes_bradti 00001000110100201020020021?0000?00?0000100010000?320211111001110010???201??1???1002111001?1?0000?122?0300???2011001101010100012?01101001103021101001?02410111??3?02210111?101?0?11??1110????010110?????????????1?00???1?????10121021???101??00?0??03[0 1]010122?02?101??101?001110

Nichollssaura_borealis 0000010011200010102010001100000100?000010010000122301010010011??211?2??0???00000?0221110101100011122?13110000?0111110??0101?112??02?1001010100100??20022100100132011?010??000??1102?101?00000?????????10110020??????????????2?12102?01?2??0000??1??200101?21012101100011101110

Pahasapasaurus_haasi 0002012?2?0?00[1 2]?1110??001??2????00?0?00????1?????22???10??????100110???????11?11?02?1000?????0?0?122?1200??????000????????????????2000211101111?100???2[1 2]1001???320???0?????0???????????????????????????????????????????????????????????2100101??111201100????1210??00011101?10

Palmulasaurus_quadratus 00?2????????00????????00???????????????????????????????????????????????????????????????????????????????????????000????????????????1000211?01????????????????????????????????????1?[1 2]?11????????01???????????????????????????0101200210022100?01??12?200101?20022101100011101211

Pantosaurus_striatus ????????????????????????????????????????????????????????????????????????????????????????????????????????????????????????????????????????????????????????100111?320??00???000100??1??110000?2000111?????????????????????????01001?021011200??10????0210101??1?1?10???211???1211

Peloneustes_philarchus 10020120211100210??010001000101?010000010020010003121100010?00101100011000011?10102211100110?010?12110200???2110110110000001111000211001020001000??21?110001?121200110100000011001201100?1?0010001?00?1011000010000002000??1002301111022000?01??000210001220022101110111111111

Picrocleidus_beloclis ?????????????????????????????????????????????????????0????0?010??0?????????????10012??????????????????????????????1100?1000???2??2?????11?????1?1101?02411011223201200111000100111?011?0?0?200?101001011002010201201020201?01?12?021112200??101???02111012210110010011111?1?10

PIMUZ_AIII0521 ??????????????????????????????????????????????????????????????????????????????????????????????????????????????0????01??0111???1??0????????????????????????????????????????????????????????????????????????????????????????????????????????????????????????????????????????????

Plesiopleurodon_wellesi 00010??0?100102?1120?000[0 1]??2?0?????????????1???222201010?10?11????????????????????????????????????????????????[0 1]001?11???111?1????1[1 2]000210?0?????????????100?0?1320?120100?0?0?0??0??110[0 1 2]???[0 1]1??101????????????20110?1?01???????????????????????????[2 3][0 1]?1012?1??????????????????

Plesiopterys_wildi 00100?0?1???00????????00???00??1?0?0???10??01?0001001000?00?0?10101110200001001010110???1????0011?00??00?100???20??100?1000?0?2??0?00?01110??????????02420011121101?001??0001001111?1100003000000?01?11?01201??0??0???010?3?0102012?001???00?000110200101?2??11001010011101110

Plesiosaurus_dolichodeirus 001100?0110?00100??0010010?000010010000100?001000100100001010110?010?02000?1111?10?01100?01000110100?000?1110?0100?100?1000???????100001110?2???0????01410010121?00100101003?00011111100001000010??211101100110000000?000?000002012100100020000?1102010000200110?1010000?01100

Plesiosaurus_manselii ?????????????????????????????????????????????????????????????????????????????????????????????????????????????????????????????????????????????1111101??2?1101???320??00?????01?01??1111?????1010011??0???????????2??1?202????????????????????????????????????????????2?????????

Pliosaurus_almanzaensis ?????????2????????????????????????????????????????????????????0?20????????????????121????1?00010?121?03?0?????1?11?01???000?12?0??????????1?????????????000????1?0???????????????????????????????????????????????????????????????????????????????????0???2????3???1?1??1??????

Pliosaurus_andrewsi 01020?2021??10210??0??00??00???????????????00?00?3?211?0??0?00??11??????????????102?11?00110?010?1211020????21111?0110000101111000211001010001001102??210001012110010000002001100120110??1?0010000??????????????????????????00230110???200??01??02021010122003210?100011011211

Pliosaurus_brachydeirus 11?1?????2??102?0??01001???0???????????????00?0???1??????????02020?????????????0?????????1?????0?12110310?????101??010?2000??13000?011010?1?0???????????0001???120??10???0?00?2010??1100????????????????????????????????????????????????0???00????0?????1?1??23???1?1??10????1

Pliosaurus_brachyspondylus ???1?????????????????????????????????????????????????????????0????????????????????????????????????????????????1011?010??000?113000????01011?010?????1?010001???120??00?????01?20??20???????????????1??1??0???0???????200?????????????????????0110?02101012100????????????????1

Pliosaurus_carpenteri 11010????2??10210??0??011?00???????????????00?00231111???20?002020??????????????????11?00110?010??2110?1??????1011??10??0?0?1110002111010111???0????1?0?0001?0?1?0?1000000200120?0??110????????????1??1?0?2000?????0?2?0???????????????2????00????1???10?????3310?1?1011?11??1

Pliosaurus_funkei ???????0?????0????????????????????????????????????????????????2?2??????????????????????????????????????????????????01????00?1????0????010?11????????????0001???1????00???0?00?2?00??1100??????????????????????2000000200?????????????????0??0?0????2?01012???3?101?0101???1??1

Pliosaurus_irgisensis ???????0??????21????????????????11?0000100200?0?231?11?002??0???2????1??????????????????????????????????????????????????????????????????????????????????000??1?1?0?1??000?200???????????????????????????????????????????????????????????001?0?????0???10??1???3???1????1?111??

Pliosaurus_cf_kevani ?????????????0???????????????????????????????????????????????????????????????????????????????????????????????????????????0????????????010?2?????????????0001???1?0??00???0?00?2?00??11??????????????0?1????????020000200???0002300111032????00??001?10101210023101101?11011211

Pliosaurus_kevani 110101?022??10210??010011000011?1100?0010020010023021100020?00202000011010?0??10?022111001100010??????????????10??0010021001113?002111010?21??????????????????????????????????????????????????????????????????????????????????????????????????????????????????????????????????

Pliosaurus_macromerus 1101????22??10210??0??011?0????????????????00?0???1??????????0???0?????????????00022111001100010?12110310?????1011??10??000?11300010110101?1??????????????????????????????????????????????????????????????????????????????????????????????????????????????????????????????????

Pliosaurus_patagonicus ???????????????????????????????????????????????????????????????????????????????????????????0?01???????????????111?0?????0???10?0????????0?1???????????????????????????????????????????????????????????????????????????????????????????????????????????????????????????????????

Pliosaurus_rossicus 11?1?1??????10210??01001??????1?11000001??200?0??3111??????????????????????????????????????????????????????????1????????0?????????1?1?01011???????????????????????????????????????????????????????????1000200?2????0?2????????????????????????????????????????????????????????

Pliosaurus_westburyensis 110101?0221110210??010011000011?1100000100200100231111000201002020???1????0????0?0121110011??????12110210???1?101?0010?200???13000?01011011?????????????0001???1?00?00?????00???00??1100??????????????????????????????????????????????????????????????????????????????????????

Polycotylus_latipinnis 00120?20?1??1021111010001110001??01?01010021???223210111010?111101????2??0???????01311111000000111?2?120?1[0 1]10?1001?110?1111?122011100011020011101002102200011113201120100100010010201110??01110000100?1000001020121012?11?1110100111113210??011?111200102221033101101011001211

QM_F51291 ????01??2?????210??010?????0210?1110000????0010???12?????????????????????????????????0?00????????????????????????????????????????????????10???????????????????????????????????????????????????????????????????????????????????????????????????????????????????????????????????

Rhomaleosaurus_cramptoni 10000021220?1020100121101?00001?1000000???0001001201100011110?10?0???1?000???????02?1110?01010110122?1000000200?11?00??2100?11???01?1?01020?00?????????200???1211000??1?0?01?1?0002110?100?0????0?????1??????????????1??????0102?11000?10???00??1?021?001?1??110110100?11?1?00

Rhomaleosaurus_thorntoni 11??0???2?0?10??1021?1101?00?????0??0001000?????????????1?????????????????????????????1??01??01?????????????200111??10?11???11000?11100101000???????????0001???110?000100??10??01?21101?00?0110100??01100120210?0?000100?10001220110001??????0111?02100002110?????????????????

Rhomaleosaurus_zetlandicus 11000??1220?102010012110??00001??000???????001001?0110?0?1110110?????1?0?0????????2211??0?1????1?1?2??0??0??2001111000021002110000??1001020?0??????????200010??110?020100?010?00??2110?1???01201000????????????????????????????????????1?0??001212021000121101101101001?????0?

Richmond_pliosaur 00010120210011201120?000[1 2]??2?0??00??00010??1???2222010101100111101?????0???????1?022??001???00?101?2?1000?0?1??001??????1???11????100011020?11101000?0210001002320?1201001000100100?1100?00111010011??1???????20?1??????????20100121011200110110111200101221022101100011101[1 2]10

Rhaeticosaurus_mertensi ????????????????????????????????????????????????????????????????????????????????????????????????????????????????11???????11?00???0?????0??0?2??????????31001[1 2]??1?0?2?0100001000??11?1000000[1 2]0??00???????????????????????????01000111000????100??1200010?1?0??1200?0?0?100????0

Sachicasaurus_vitae 01?2??2121??10210??01010?00?2?0?1?10?0?1?1?00?1??31201??1?0?00?????0???????0????????10?0?01110010?21?03??0002?110001????011?11?0000011010?00001?01?0??10000?1??321?2000?000?0?001?10000????1010000????1?0020?00?????????????0?03011?103??????0??1??110??1?111????????????????1

Seeleyosaurus_guilelmiimperatoris 00000?00?10?02?????0???????0?????0??0011001????1?1?01000?0??11?????????????????????????????????????????????????10??100?1010???????100001100?2??????????3100?1221000???1???00?00?1200110010?100001101101001201?10030?1?0???300?02??200011000100??11020?100?20011011000011201101

Simolestes_vorax 110101???21?10210??010001??010??0100000100200?0003111?000?0?0010110??11000????1???221110???????0?121?0210???210111?110?0000?111000??1?01010?0??00???111100011121?0?1000001200110????11?????0????0??20?100120101020000?0????000230??1102201??010100021010122001110111001111????

Speeton_clay_plesiosaurian ????????????????????????????????????????????????????????????????????????????????????????????????????????????????????????????????????????????2???????????2101012320120011110010011201111000?20?1111??1011?1??20??1??002?????010121011011200??00102103001022?1021001100011101?11

Spitrasaurus_spp ??????0?????0?????????????????????????????????????????0???00??1010?????????????1??????????????????0???????????0201?100?1000???2??0?00?01110?20111111??2521012213201200111030100110?01100??????????????11?0???0?0??01???2???????????????2?11?????1?02?0?012?1???101102?1???1?11

Stenorhynchosaurus_munozi 00020?21220?00210??010010000110?11100001?1?0001?0312???01?0??0??1?1??1?????0??????1?10?0011010010021103?10002110000010?0001?1120?20001010220011?0100??1100021??3201210????001?0???2????????????????20?1???2000??200??2??????????????10???02100000101????1?0??33101102001211?01

Stratesaurus_taylori 001000?0100100100??0110010000010?0?000010000010001001000010101101012002000010?00102211???01000010110?000000?2?111?11000110001?0?001000011000?0?00??2001?10010011100120100003101??1????00?0????????????1?????????????????????01022111??????????????????????????1???010??010??10

Styxosaurus_snowii 00001??0?100002?12200?0020?0?0??00??00010011?????3??111110?001??????????????????????????????????????????????20010[0 1]100112000?112?001110011031????111??0??211?1??3?022201???1???0???????????????????????????????????????????????????????????????????????????????????????????????

Sulcusuchus_erraini ?0?[1 2]?1?0?10???21112?100?1??2????0??????????1???????0??????0??1??01?????0???????10022?????????????122?1?01?????100??110??101?1?2011??0?21??0???????????????????????????????????????????????????????????????????????????????????????????????????????????????????????????????????

Tatenectes_laramiensis ???????0?????????????????0??????0??????1????????????2000?0000??????????????????????????????????11????????0?1???????????????????????????11??????01001????00011??32011001?1000100?110?110000??????????1011???????01??1020101?10?110021011210??10????0210101??1?2?001?0211??01??1

Terminonatator_ponteixensis 000010?01?0000201020??002??00?0?001????10??1????0[2 3]21??[0 1]11??001?????????????0010?????????1???0??????????????????100?10111110??12?0010100110312??????????53111222320?20011111010011??????????100???1??????????????????????????1?12??1??1?100??00001103101012210331011010110?1211

Thalassiodracon_hawkinsii 00100001110100111120??0?1000???0000000?10020000001011000010110101010002000011000101011?00011001??100?000????0?11101100010000?1200?001001110010000??2101310010011100120100002100011101000001011000002011011001?00000001000?0001020111001000200000110100000010?11001010000101100

Thalassomedon_haningtoni_composite_SK 00001000??0010211120000020?000010010000100111???0200??011?0?010?10????2????????1??221????????????1?2?1??????20??01010???001?1????00010011?302010110210262111022320221011011?0000111011001002000100???01?002020??2??00111001121202021[0 1]11000???010110[2 3]10100220111111012[0 1]1101?210

Thililua_longicollis 000210?0?10000201110000001?2000?001000010??1????2221??111?0?11????????????????????????????????????????????????10000?1???1???02????1000110101[1 2]0??1[0 1]0??0?3110110132011201000100?00???01?????????????????????????????????????????????????????????????????????????????????????????

Tricleidus_seeleyi 01100???????011010?00?0010?000?100?????????01001?3001000?000010?00?0111111?????111110?????????011200?000?1100?0201110001000?0021?01110011001?11?1101?02210011123201100111000100?11?01100???????????110110000002012010202?13????????????210??1011130210101221023001101111101211

Trinacromerum_bentonianum 00020121?1?000211110100011?0001??01?01010021???1?31?11110???01??0????12010??????001311111??0???11122?13001?00??001?11???10??12???1?000211200?1101000?02?00012??3201[0 1]201000?00?00????11??????1?000???11101120?0201010020110111?110?211?32????0?????1???10?2???33?0??01011001211

Tuarangisaurus_keyesi 000010?0??0000??1020020020?000???0??0001??11????032?2111?00001??????????????????????11?????????0??2????????????10??10??1010???2??010000110?1?0111111?02?21111??3?022?0111?101?0???????????????????????????????????????????????????????????????????????????????????????????????

Umoonasaurus_demoscyllus 00000100112??1101020??00??00????0??????????0?002222?10???10?11????1??12?11?0??0??022?????0?????11122?1211000????????0??1111???????1111010?01????????????1001????20?1??1010??00?110101102000201?00?1???1?01?010?????????????0???????????21????0111?02001022210?2???1?0??10?1??1

Vectocleidus_pastorum ??????????????????????????????????????????????????????????????????????????????????????????????????????????????????????????????????????????????????????????01?01??0?1??1010??00??10??1102?????????????1???1?02???1??01?10?0????????????????????????????????????????????????????

Vegasaurus_molyi ?????????????????????????????????????????????????????????????????????????????????????????????????????????????????????????????????????????????11?1101?0252111?2232022101111?0100???10111000?2011101?20?1100111?212001021????010111011000?0???000?1?0310111231022???001??1?01???

Wapuskanectes_betsynichollsae ?????????????????????????????????????????????????????????????????????????????????????????????????????????????????????????????????????????????????????????????????????????????????1??1??0???????????2101?2?110?1110110?10??3????????????200???01???03000022???????????????????0

Westphaliasaurus_simonsensii ????????????????????????????????????????????????????????????????????????????????????????????????????????????????????????????????????????????2????????????0011021?0??0010?1031000111?1?0010?0000101?211100120??00000001??10000??20?20001?????00011002100011100110110100011?1??1

Zarafasaura_oceanis 0000100011000020102002002000000?001100011021???0020[0 1]21110000010?010??12?1??0??0??0?????010100010?12?????0?????0101010111000??[1 2]2?001100011030??10?????02?1101022320?200111110000111[0 1]?1110???20???01?2?01?01111?11100?0??????11?11?021001?0???00????03101112???[1 2]???1?0000???????

;

ccode + 3 6 8 9 14 21 24 27 28 37 42 47.49 51 52 62 64 67 68 70 83 98 102 111 125 130 136 137 140 150.152 158.160 163 174 177 178 183 186 187 189 195 204 206 207 223 226 230 231 233 234 240 241 243 244 248.250 253 254 260 261 264 267 *;

proc /;

comments 0

;
