# Supplementary material for: The rise of macropredatory pliosaurids near the Early-Middle Jurassic transition
Source: Sci Rep. 2023 Oct 16;13:17558. doi: 10.1038/s41598-023-43015-y (PMC10579310; doi:10.1038/s41598-023-43015-y)
Supplement: Supplementary file 3 — Supplementary Information 3. [file 41598_2023_43015_MOESM3_ESM.docx]

Electronic Supplementary Material 3 for:

**The rise of macropredatory pliosaurids near the Early-Middle Jurassic transition**

Sven Sachs, Daniel Madzia, Ben Thuy and Benjamin P. Kear

**Table S1.** Data for the multivariate analyses of Plesiosauria

| TAXON | C1 | C2 | C3 | C4 | C5 | C6 | C7 | C8 | C9 | C10 | C11 |
| --- | --- | --- | --- | --- | --- | --- | --- | --- | --- | --- | --- |
| *Marmornectes candrewi* | 25.3 | 2.976470588 | 0 | 1 | 0 | 0 | 0 | 1 | 1 | 0 | 1 |
| *Pachycostasaurus dawni* | 33 | 1.736842105 | 0 | 1 | 1 | 0 | 0 | 0 | 1 | 0 | NA |
| ‘*Pliosaurus*’ *andrewsi* | 41.1 | 1.763948498 | 0 | 0 | 0 | 0 | 1 | 1 | 0 | 0 | 2 |
| *Peloneustes philarchus* | 31.3 | 2.576131687 | 0 | 1 | 0 | 0 | 0 | 1 | 0 | 0 | 1 |
| *Simolestes vorax* | 82.4 | 2.559006211 | 0 | 1 | 0 | 0 | NA | 0 | 1 | 2 | 1 |
| *Liopleurodon ferox* | 81 | 2.436090226 | 0 | 1 | 0 | 0 | NA | 1 | 0 | 1 | 2 |
| *Anguanax zignoi* | 18 | 1.5 | NA | NA | NA | 0 | 0 | 2 | NA | NA | NA |
| *Gallardosaurus itturraldei* | NA | NA | 1 | 0 | NA | 1 | 0 | NA | NA | NA | NA |
| *Pliosaurus brachydeirus* | NA | 2.2 | 2 | 1 | 0 | 2 | 1 | 2 | 0 | 1 | 0 |
| *Pliosaurus kevani* | 48 | 1.655172414 | 1 | 1 | 0 | 1 | 1 | NA | 0 | 1 | NA |
| *Pliosaurus westburyensis* | 110 | 2.2 | 2 | 1 | 0 | 2 | 1 | 2 | 0 | 1 | 0 |
| *Pliosaurus carpenteri* | 100 | 2 | 2 | 1 | 0 | 2 | 1 | 2 | 0 | 1 | 0 |
| ‘*Pliosaurus*’ *rossicus* | 130 | 3.25 | 2 | 0 | 0 | 2 | 1 | 2 | 0 | 1 | 0 |
| ‘Kheta pliosaurid’ | 63 | 1.96875 | 0 | 0 | 0 | 0 | 0 | 0 | 1 | 0 | 1 |
| ‘Maryevka pliosaurid’ | 28 | 1.866666667 | 0 | 1 | 1 | 0 | 0 | 0 | 2 | 1 | 1 |
| ‘Rudnichnyi pliosaurid’ | 75 | 1.88 | 0 | 1 | 0 | 0 | 0 | 0 | 0 | 1 | 2 |
| ‘Crimean pliosaurid’ | 22 | 1.466666667 | 2 | 0 | 0 | 2 | 1 | 2 | 0 | 0 | NA |
| *Makhaira rossica* | 48 | 3 | 3 | 1 | 0 | 2 | 1 | 2 | 0 | 0 | NA |
| *Luskhan itilensis* | NA | NA | 1 | NA | 0 | 1 | 1 | 2 | 0 | 0 | NA |
| *Acostasaurus pavachoquensis* | NA | NA | 0 | 1 | 0 | 0 | 0 | 0 | 2 | 0 | NA |
| *Stenorhynchosaurus munozi* | NA | NA | 1 | 1 | 0 | 1 | 1 | 2 | 0 | 0 | 0 |
| *Kronosaurus queenslandicus* | 100 | 2 | 0 | 0 | 0 | 0 | 0 | 0 | 2 | 0 | NA |
| *Monquirasaurus* *boyacensis* | 90 | 2.5 | 0 | 1 | 0 | 0 | 0 | 0 | 0 | NA | 1 |
| *Megacephalosaurus eulerti* | 89 | 1.854166667 | 0 | 0 | 1 | 0 | 0 | 0 | 0 | 0 | 0 |
| *Brachauchenius lucasi* | 25 | 2 | 0 | 1 | 1 | 0 | 0 | 0 | 0 | 0 | NA |
| ‘*Polyptychodon*’ type 1 | 70 | 2 | 0 | 0 | 0 | 0 | 0 | 1 | 0 | 0 | 1 |
| ‘*Polyptychodon*’ type 2 | 70 | 2 | 0 | 0 | 0 | 0 | 0 | 0 | 2 | 0 | 1 |
| ‘*Polyptychodon*’ type 3 | 95 | 1.9 | 0 | 1 | 0 | 0 | 0 | NA | 0 | 1 | NA |
| ‘Annopol pliosaurid’ | 36 | 1.8 | 0 | 0 | 0 | 0 | 1 | 0 | 0 | 0 | 1 |
| *Lorrainosaurus keileni* | 30 | 1.9 | 0 | 1 | 1 | 0 | 0 | 0 | 2 | 1 | 1 |
| *Maresaurus coccai* (Bajocian) | 25 | 1.47 | 0 | NA | 0 | 0 | 0 | 1 | 1 | 0 | NA |
| *Borealonectes russelli* (Callovian) | NA | NA | 0 | 1 | NA | 0 | 0 | NA | NA | NA | NA |
| *Anningasaura lymense* (Hettangian-Pliensbachian) | 20 | 3.13 | 0 | NA | 0 | 0 | 1 | NA | 0 | 0 | 0 |
| *Meyerasaurus victor* (Toarcian) | 29 | 3.2 | 0 | NA | 0 | 0 | 0 | 2 | NA | 0 | NA |
| *Archaeonectrus rostratus* (Sinemurian) | NA | NA | 0 | NA | 0 | 0 | 0 | 1 | 2 | 0 | 0 |
| *Rhomaleosaurus cramptoni* (Toarcian) | NA | NA | 0 | NA | 0 | 0 | 0 | 2 | 0 | 0 | NA |
| *Rhomaleosaurus thorntoni* (Toarcian) | NA | NA | 0 | 1 | 0 | 0 | 1 | 2 | 0 | 0 | NA |
| *Rhomaleosaurus zetlandicus* (Toarcian) | NA | NA | 0 | 1 | 0 | 0 | 0 | 2 | 0 | 0 | 0 |
| *Thaumatodracon wiedenrothi* (Sinemurian) | 11 | 2.2 | 0 | NA | 0 | 0 | 1 | 2 | 2 | 0 | 1 |
| *Macroplata tenuiceps* (Hettangian) | 16 | 2 | 0 | NA | 0 | 0 | 0 | 2 | 2 | 0 | NA |

**Sources used to score rhomaleosaurid taxa**

*Maresaurus coccai* [1], *Borealonectes russelli* [2], *Anningasaura lymense* [3], *Meyerasaurus victor* [4, pers. obs.], *Archaeonectrus rostratus* [5], *Rhomaleosaurus cramptoni* [6], *Rhomaleosaurus thorntoni* [7], *Rhomaleosaurus zetlandicus* [8, 9], *Thaumatodracon wiedenrothi* [10], *Macroplata tenuiceps* [11].

**References**

1. Gasparini Z. A new pliosaur from the Bajocian of the Neuquén basin, Argentina. *Palaeontol.* **73**, 677–681. (1997)

2. Sato T, Wu XC. A new Jurassic pliosaur from Melville Island, Canadian Arctic Archipelago. *Can. J. Earth Sci.* **45**, 303–320. (2008)

3. Vincent P, Benson RBJ. *Anningasaura*, a basal plesiosaurian (Reptilia, Plesiosauria) from the Lower Jurassic of Lyme Regis, United Kingdom. *J. Vertebr. Paleontol*. **32**, 1049–1063. (2012)

4. Smith AS, Vincent P. A new genus of pliosaur (Reptilia: Sauropterygia) from the Lower Jurassic of Holzmaden, Germany. *Palaeontol.* **53**, 1049–1063. (2010)

5. Owen R. A monograph on the fossil Reptilia of the Liassic Formations. Part 3. Sauropterygia. Monographs Of The Palaeontographical Soc. **17**, 1–40. (1865)

6. Smith AS. Anatomy and Systematics of the Rhomaleosauridae (Sauropterygia: Plesiosauria). National University of Ireland, University College Dublin. Unpublished PhD thesis. (2007)

7. Smith AS, Benson RBJ. Osteology of *Rhomaleosaurus Thorntoni* (Sauropterygia: Rhomaleosauridae) from the Lower Jurassic (Toarcian) of Northamptonshire, England. *Palaeontogr. Soc. Monogr.* **168**, 1–40. (2014)

8. Taylor MA. Functional anatomy of the head of the large aquatic predator *Rhomaleosaurus zetlandicus* (Plesiosauria, Reptilia) from the Toarcian (Lower Jurassic) of Yorkshire, England. *Philos. Trans. R. Soc. Lond. B* **335**, 247–280. (1992)

9. Vincent P, Smith AS. A redescription of *Plesiosaurus propinquus* Tate & Blake, 1876 (Reptilia, Plesiosauria), from the Lower Jurassic (Toarcian) of Yorkshire, England. *Proc. Yorkshire Geol. Soc.* **57**, 133–142. (2009)

10. Smith AS, Araújo R. *Thaumatodracon wiedenrothi*, a morphometrically and stratigraphically intermediate new rhomaleosaurid plesiosaurian from the Lower Jurassic (Sinemurian) of Lyme Regis. *Palaeontographica A* **308**, 89–125. (2017)

11. Ketchum HF, Smith AS. The anatomy and taxonomy of *Macroplata tenuiceps* (Sauropterygia, Plesiosauria) from the Hettangian (Lower Jurassic) of Warwickshire, United Kingdom. *J. Vertebr. Paleontol*. **30**, 1069–1081. (2010)
