# Supplementary material for: The rise of macropredatory pliosaurids near the Early-Middle Jurassic transition
Source: Sci Rep. 2023 Oct 16;13:17558. doi: 10.1038/s41598-023-43015-y (PMC10579310; doi:10.1038/s41598-023-43015-y)
Supplement: Supplementary file 6 — Supplementary Figures. [file 41598_2023_43015_MOESM6_ESM.docx]

Electronic Supplementary Material 6 for:

**The rise of macropredatory pliosaurids near the Early-Middle Jurassic transition**

Sven Sachs, Daniel Madzia, Ben Thuy and Benjamin P. Kear

**
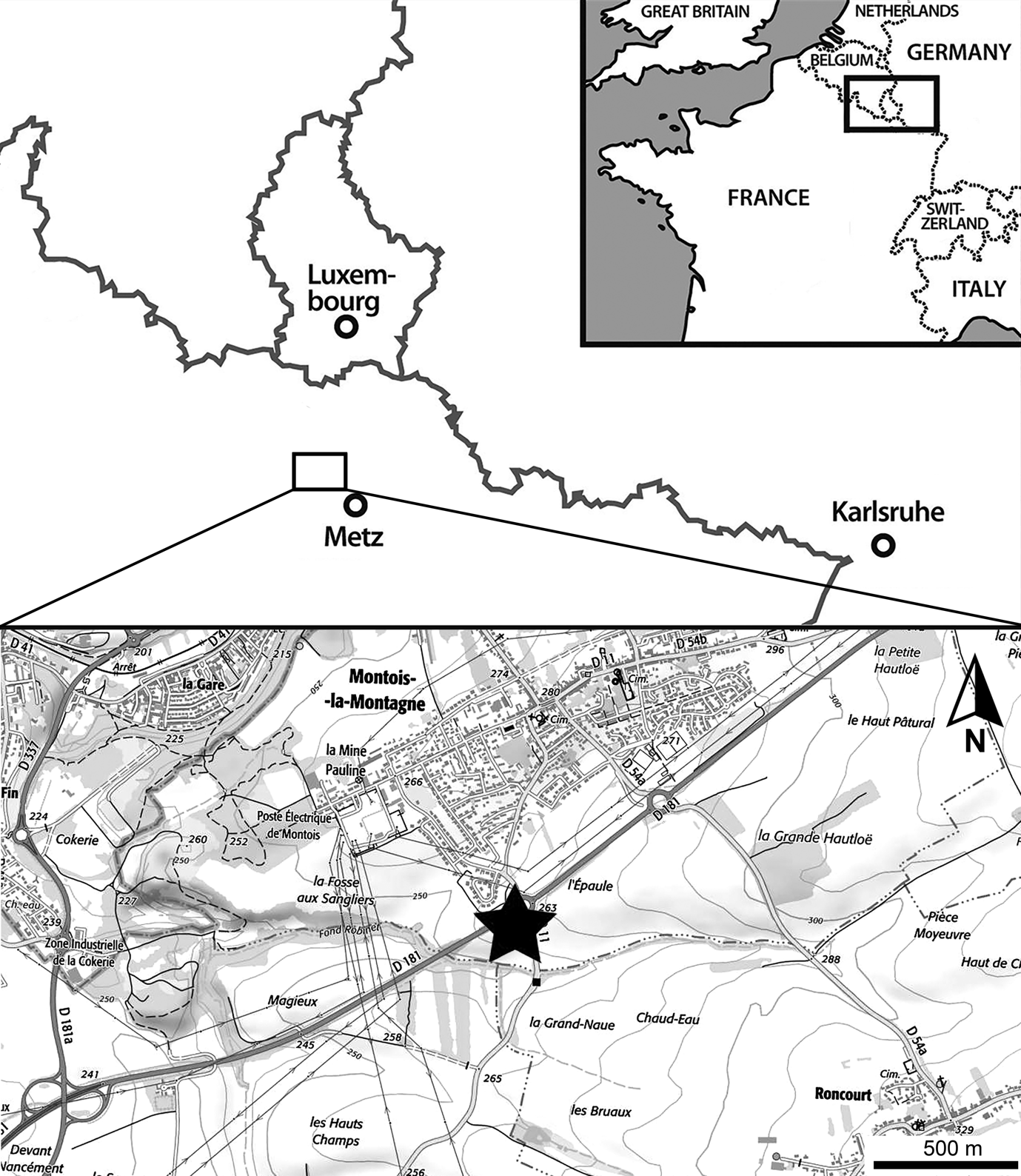
**

**Figure S1**. Map showing the source locality of MNHNL BU159 (black star) along the road between Montois-la-Montagne and Sainte-Marie-aux-Chênes (bottom), northeast of Metz in Lorraine, northeastern France (top and right insert).


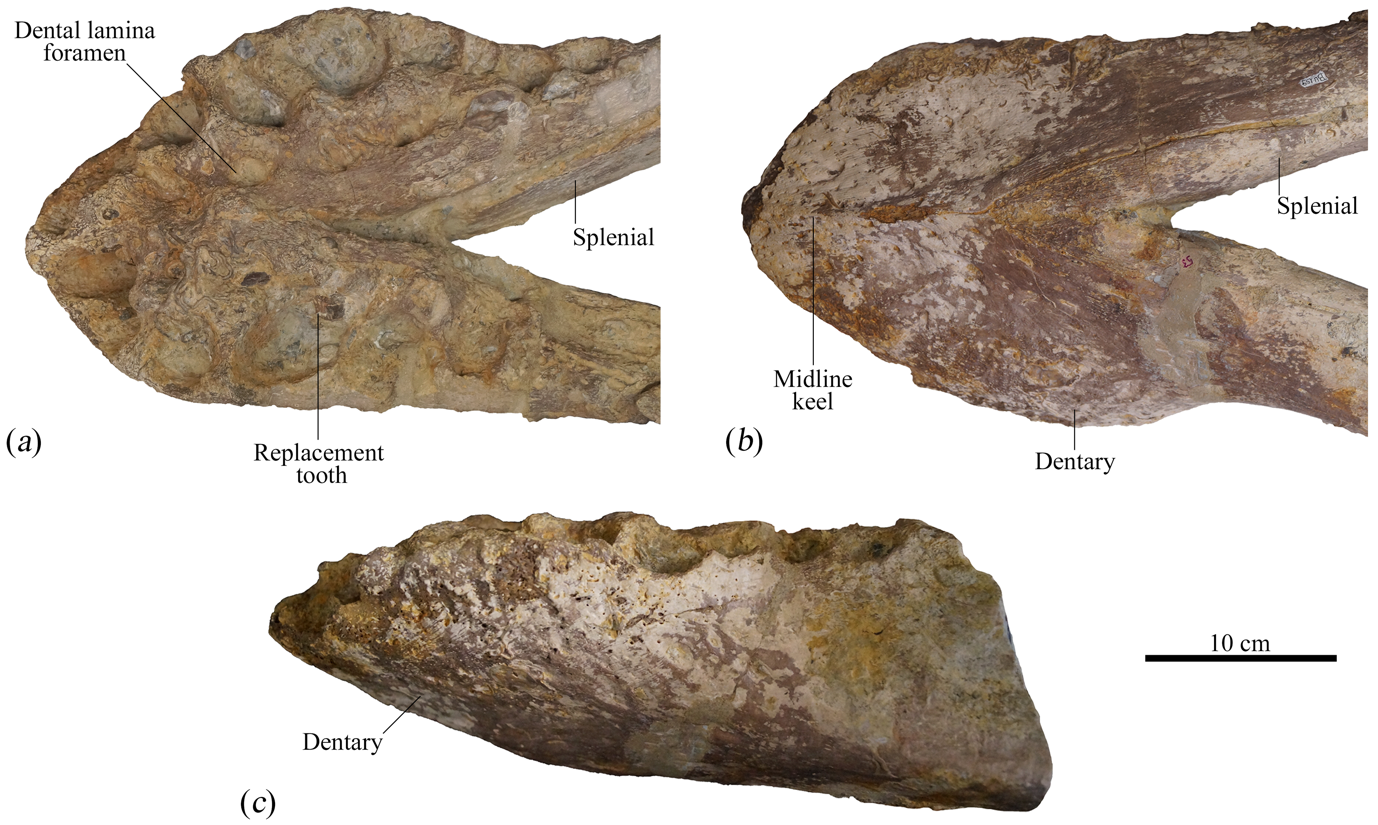


**Figure S2**. Symphyseal section of the mandible from the holotype (MNHNL BU159) of *Lorrainosaurus keileni* in (*a*) dorsal, (*b*) ventral, and (*c*) lateral views.


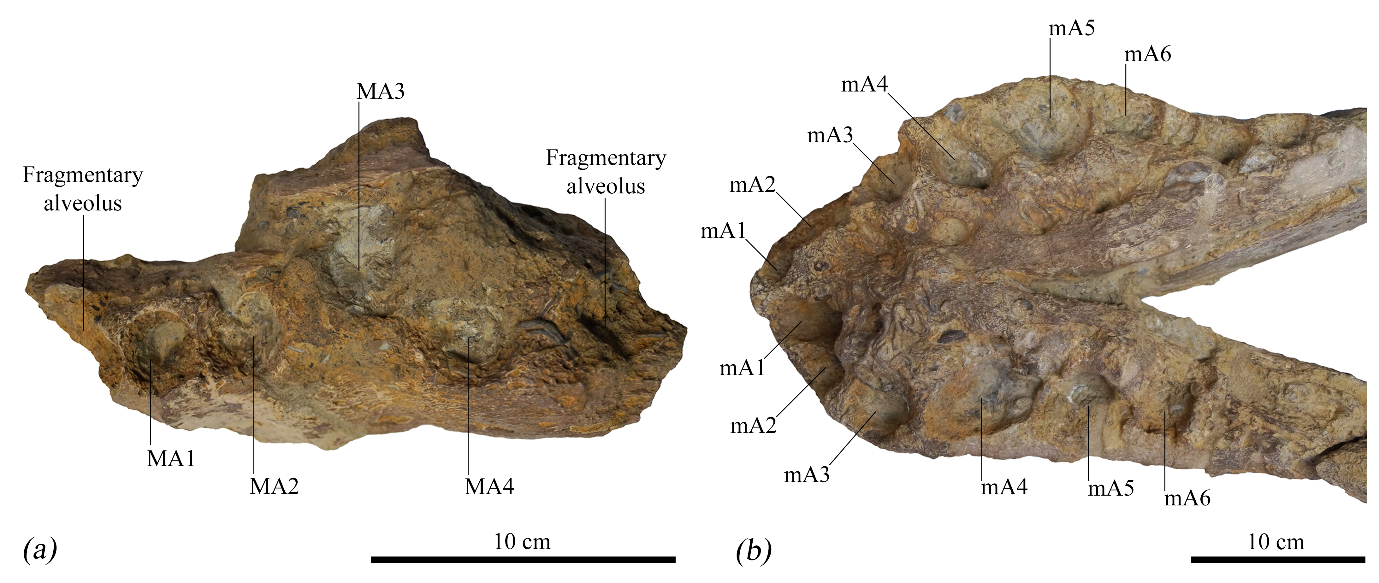


**Figure S3.** Tooth positions in (*a*) the maxillary fragment (MA) and (*b*) the symphyseal section of the mandibular rostrum (mA) in the holotype (MNHNL BU159) of *Lorrainosaurus keileni*. See figures 2 and 3 in the main text for more information.

**
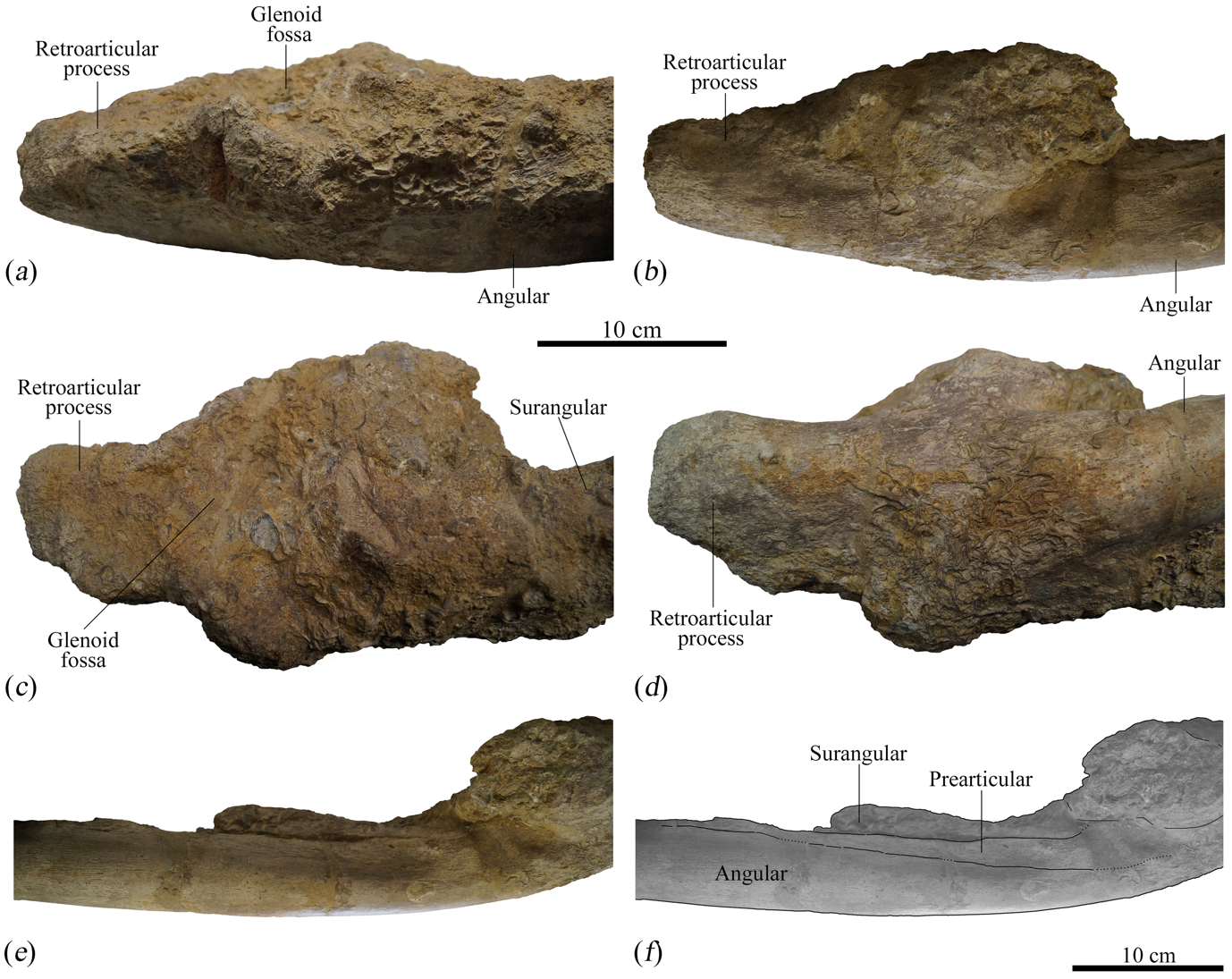
**

**Figure S4**. Enlargements of the posterior section of the mandible from the holotype (MNHNL BU159) of *Lorrainosaurus keileni.* Glenoid region and retroarticular process in (*a*) lateral, (*b*) medial, (*c*) dorsal, and (*d*) ventral views. Prearticular section in medioventral view: (*e*) photograph; (*f*) graphic illustrating individual bones and important structures.


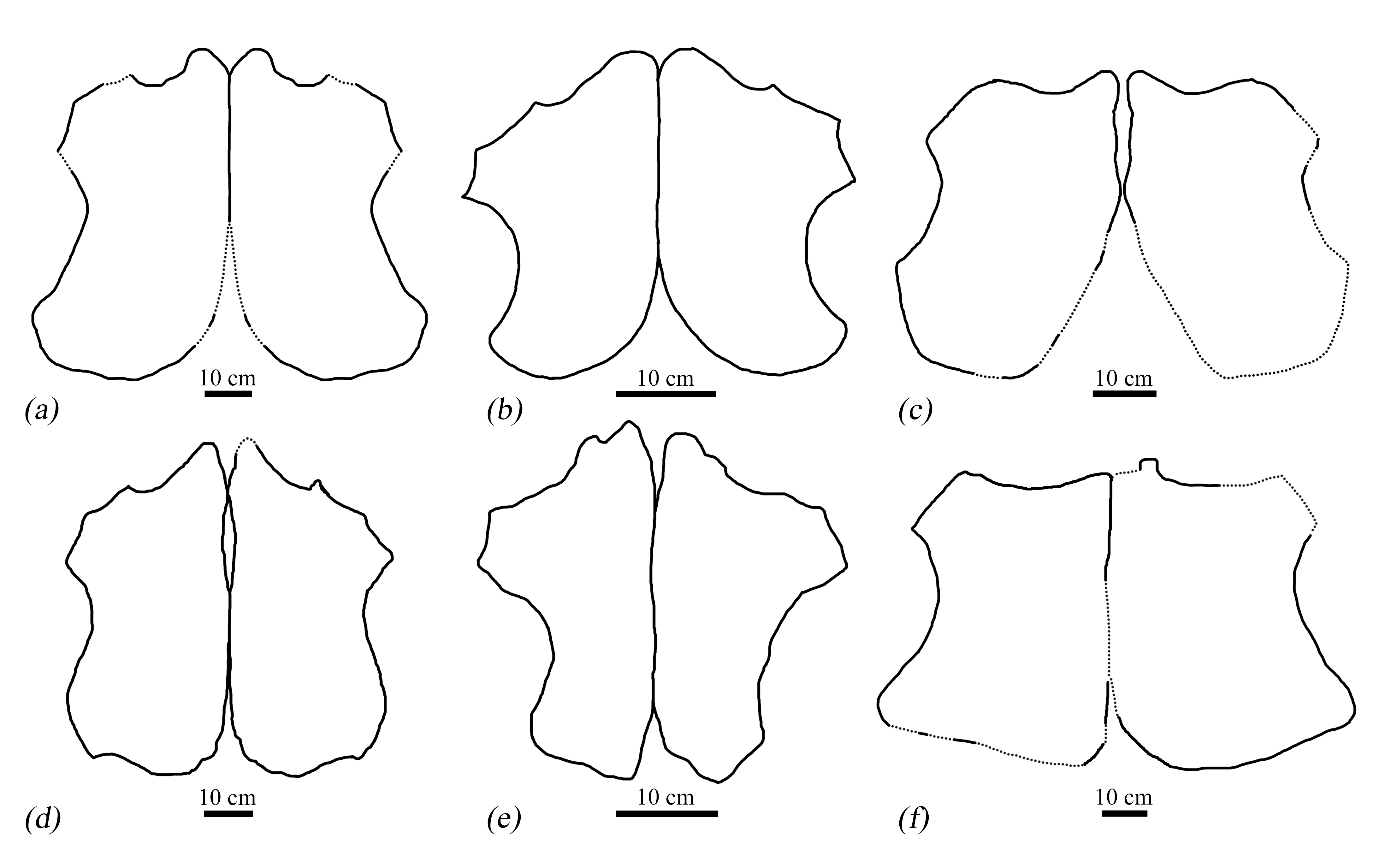


**Figure S5**. Comparison of coracoid morphologies, showing posteromedially widely separated (*a*-*c*) and only slightly split (*d-e*) coracoids. (*a*) *Lorrainosaurus keileni* (reconstruction based upon the preserved left coracoid), (*b*) *Attenborosaurus conybeari* (after Sollas 1881, pl. 23, fig. 3), (*c*) *Brachauchenius* cf. *lucasi* (after Albright et al. 2007, fig. 10), (*d*) *Simolestes vorax* (after Andrews 1913, text-fig. 8B), (*e*) *Hauffiosaurus zanoni* (after Vincent 2011, fig. 5A), (*f*) *Luskhan itilensis* (after Fischer et al. 2023, Fig. 10J, K).
